# HAI-2 as a novel inhibitor of plasmin represses lung cancer cell invasion and metastasis

**DOI:** 10.1038/s41416-019-0400-2

**Published:** 2019-02-15

**Authors:** Shang-Ru Wu, Chia-Hao Lin, Han-Po Shih, Chun-Jung Ko, Hsin-Ying Lin, Shao-Wei Lan, Hsin-Hsien Lin, Hsin-Fang Tu, Chao-Chi Ho, Hsiang-Po Huang, Ming-Shyue Lee

**Affiliations:** 1 Department of Biochemistry and Molecular Biology, College of Medicine, National Taiwan University, R817, 8F, No. 1, Section 1, Jen-Ai Rd., Taipei, Taiwan; 2 Department of Immunology, the University of Texas MD Anderson Cancer Center, Taipei, Taiwan; 3 Department of Internal Medicine, National Taiwan University Hospital, College of Medicine, National Taiwan University, Taipei, Taiwan; 4 Graduate Institute of Medical Genomics and Proteomics, College of Medicine, National Taiwan University, Taipei, Taiwan

**Keywords:** Non-small-cell lung cancer, Metastasis, Cell invasion

## Abstract

**Background:**

Dysregulation of pericellular proteolysis usually accounts for cancer cell invasion and metastasis. Isolation of a cell-surface protease system for lung cancer metastasis is an important issue for mechanistic studies and therapeutic target identification.

**Methods:**

Immunohistochemistry of a tissue array (*n* = 64) and TCGA database (*n* = 255) were employed to assess the correlation between serine protease inhibitors (SPIs) and lung adenocarcinoma progression. The role of SPI in cell motility was examined using transwell assays. Pulldown and LC/MS/MS were performed to identify the SPI-modulated novel protease(s). A xenografted mouse model was harnessed to demonstrate the role of the SPI in lung cancer metastasis.

**Results:**

Hepatocyte growth factor activator inhibitor-2 (HAI-2) was identified to be downregulated following lung cancer progression, which was related to poor survival and tumour invasion. We further isolated a serum-derived serine protease, plasmin, to be a novel target of HAI-2. Downregulation of HAI-2 promotes cell surface plasmin activity, EMT, and cell motility. HAI-2 can suppress plasmin-mediated activations of HGF and TGF-β1, EMT and cell invasion. In addition, downregulated HAI-2 increased metastasis of lung adenocarcinoma via upregulating plasmin activity.

**Conclusion:**

HAI-2 functions as a novel inhibitor of plasmin to suppress lung cancer cell motility, EMT and metastasis.

## BACKGROUND

Non-small cell lung carcinoma (NSCLC) is a primary type of lung cancer with approximately 85% of the incidence.^1^ The lung cancer patients with metastasis have less than 5% of the five-year survival rate.^2^ The dysregulation of pericellular proteolysis in the tumour environment often occurs to be a main factor for extracellular matrix (ECM) degradation and cancer cell movement, ultimately contributing to metastasis.^3,4^ Therefore, understanding the molecular mechanisms how metastatic cells abuse pericellular proteolysis will provide insights for the identification of therapeutic targets, as well as development of novel strategies against lung cancer.

Hepatocyte growth factor activator inhibitor-2 (HAI-2) is a bi-Kunitz-type serine protease inhibitor containing two Kunitz domains (KD1 and KD2), first identified in placenta and gastric carcinoma cells with a homology to HAI-1, and has an inhibitory activity against hepatocyte growth factor activator (HGFA).^5–7^ HAI-2 also represses the other serine proteases including kallikrein and coagulation factor XIa.^8^ Several lines of evidence indicate that down-regulation of HAI-2 is involved in cancer progression; for examples, the low expression or genetic silencing of HAI-2 has been found in hepatocellular carcinoma (HCC), prostate cancer, melanoma and gastric cancer.^9–12^ Moreover, HAI-2 is capable of suppressing cancer cell invasion and metastasis, including HCC, melanoma, prostate cancer and endometrial cancer.^9,11–13^ The anti-invasive and anti-metastatic abilities of HAI-2 result from suppression of several serine proteases, such as matriptase and hepsin.^11,14^

The urokinase-plasminogen activation system (uPAS) is a pericellular serine protease cascade that plays crucial roles in ECM remodeling for tissue development and regeneration, even for cancer cell invasion and metastasis.^15^ The components of uPAS include plasminogen and urokinase-type plasminogen activator (uPA), and their cognate Serpin-type inhibitors (PAI-1 and PAI-2).^15,16^ During cancer progression, uPA proteolytically activates plasminogen into plasmin and is a primary trigger for the activation of uPAS.^17^ Subsequently, plasmin has a feedforward effect to activate its activator zymogen (pro-uPA), such that a reciprocal activation loop accelerates the plasminogen activation.^18^ In addition to pro-uPA, plasmin has several substrates (fibrin, fibronectin, thrombospondin, laminin, collagenases, growth factors, etc.) to function as a powerful protease for fibrinolysis, tissue remodelling and cancer progression.^19^ Plasmin is often aberrantly turned on in malignancy for invasive tumour growth and dissemination.^20^ The uPAS can activate other pericellular proteases to promote cancer progression. For example, plasmin has been shown to induce the proteolytic activation of metalloproteases-2 and 9 (MMP-2 and MMP-9).^21^ Moreover, uPAS triggers the proteolytic activation of growth factors, including hepatocyte growth factor (HGF) and transforming growth factor-β1 (TGF-β1), to promote malignancy.^22–24^ The expression levels of uPA and uPAR are up-regulated in NSCLC compared to normal tissues.^25,26^ The recent meta-analysis of clinical patients’ archival specimens have also suggested that uPA/uPAR are clinical biomarkers for NSCLC.^27^

Epithelial-mesenchymal transition (EMT) is a programmed process for cell transformation from epithelial to mesenchymal phenotypes, making cell polarity lost and cell motility increased to promote metastasis.^28^ During EMT process, the loss of E-cadherin is often accompanied with the up-regulation of mesenchymal neuron cadherin (N-cadherin) or a cytoskeleton protein Vimentin.^29,30^ EMT program is controlled by specific transcription factors, including Snail, Slug, ZEB (Zinc-E-box binding), and Twist.^31^ Among them, Slug has been reported to play an important role in EMT of NSCLC, promoting cancer cell invasion and drug resistance.^32^

Although HAI-2 was described in many cancers, it still remains elusive if HAI-2 plays a role in lung cancer. In this study, we aimed to investigate the action of HAI-2 on lung cancer progression by identifying HAI-2’s target protease(s) and to delineate the mechanism how HAI-2 mediates a protease system in NSCLC. Our findings showed that the expression level of HAI-2 in the archival specimens of lung cancer decreased following the NSCLC progression. HAI-2 overexpression repressed NSCLC cell motility. We identified plasmin(ogen) as an in vivo target of HAI-2, and demonstrated that HAI-2 serves as a novel non-covalent inhibitor for uPAS system by targeting cell-surface plasmin. In addition, HAI-2 could regulate the EMT program of NSCLC partly via suppressing the plasmin-mediated activation of pro-HGF and pro-TGF-β. Our results together highlight the unique role of HAI-2 in NSCLC cell invasion, EMT and metastasis through repressing plasmin.

## Materials and methods

For full descriptions of materials and methods, please see Supplementary Materials and Methods.

### Cell Culture

Human lung adenocarcinoma cell lines were maintained in RPMI 1640 supplemented with 10% FBS and 1% L-glutamine.

### Lentiviral infection for shRNA interference and overexpression

The lentivirus vector system utilised to deliver the shRNA and gene of interest was purchased from the RNAi Core Facility (Academia Sinica, Taipei, Taiwan) The protocols for viral particle production and infection were provided by the RNAi Core Facility.

### Recombinant protein purification

GST fusion proteins were produced in BL21 (DE3) *E.coli* using IPTG induction for 4 h. After cell lysis, the lysates were collected and the GST fusion proteins are purified by affinity columns. The titerless amplification of viral particles and production of recombinant proteins were referred to the previous study.^33^

### Animal models and lung metastatic assay

NOD/SCID mice (6 months old) were obtained from the National Laboratory Animal Center (Taipei, Taiwan) and breed following the animal use protocol of IACUC, Academia Sinica. Cells expressing Luc2 gene were suspended at a density of 1 × 10^6^ cells per 100 µl OPTI-MEM and intravenously injected into the tail vein of each mouse.

### Statistics

The statistical results were calculated from three independent experiments. The significance was determined by one-way ANOVA or Student’s *t*-test using Prism 6 software (GraphPad, CA, USA).

## Results

### Inverse correlation of HAI-2 expression with lung cancer progression and poor prognosis

**Fig. 1:**
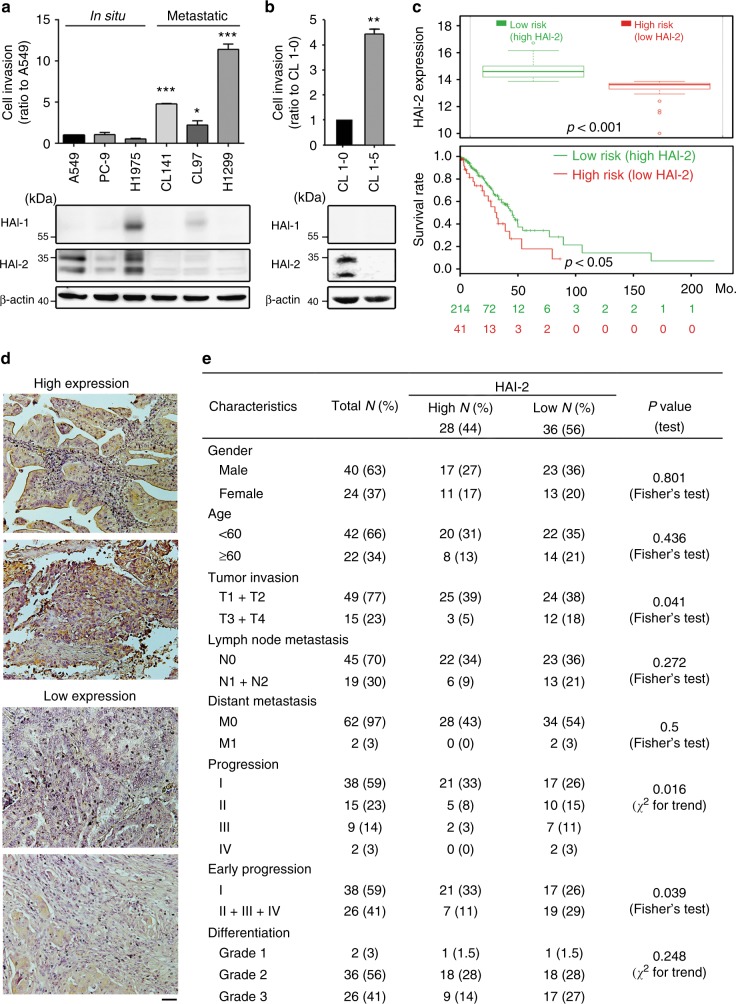
Down-regulation of HAI-2 is related to the invasion, survival rate and progression of NSCLC. Analyses of the cell invasion and the expression of HAI-1 and HAI-2 in different lung cancer cells (**a**) and in lowly invasive CL1-0 and highly invasive CL1-5 cells (**b**). The cells penetrating through the transwells were represented as the migrating or invasive abilities. (mean ± S.D., *n* = 3; *p < 0.05; **p < 0.005; ***p < 0.001). **c** Association of survival rate and HAI-2 expression in lung cancer patients. The tissue samples of 255 lung adenocarcinoma patients (SurvExpress, #13 TCGA database) were divided into High risk and Row risk groups according to the HAI-2 expression levels (upper panel). The survival rates of patients in High risk and Low risk groups were shown in the lower panel. **d** Immunohistochemical images of HAI-2 in lung adenocarcinoma (64 cases, LC641, Biomax) are divided into two groups: high HAI-2 expression (*n* = 28) and low HAI-2 expression (*n* = 36). Scare bar = 100 μm. Two representative images from each group were shown. **e** Examination of the associations between HAI-2 protein levels in the IHC images (**d**) and cancerous characteristics. The significant correlation was statistically determined by Fisher’s Exact test or *χ*^2^ test

To investigate the roles of HAI-1 and HAI-2 in the progression of lung adenocarcinoma, we first analysed the levels of HAI-1 and HAI-2 among six lung adenocarcinoma cell lines and divided them into two groups according to their originality. Among them, A549, PC-9 and H1975 lung adenocarcinoma cells were originally isolated from in situ lung cancer lesions and represent the non-metastatic group of lung adenocarcinoma; pleural effusion-derived cells (CL141 and CL97) and lymph node-derived cells (H1299) are considered to be the metastatic group of lung adenocarcinoma.^34–37^ The results from cell invasion assays showed that the cells in the metastatic group had highly invasive abilities (Fig. 1a, upper panel) and expressed low levels of HAI-2, compared to those in the non-metastatic (in situ) group (Fig. 1a, lower panel). HAI-2 exhibits two major bands by western blot due to its N-glycosylation.^38,39^ HAI-1 expression was only present in H1975 and CL97 cells and showed no correlation with the cell invasion (Fig. 1a, lower panel). Moreover, we examined the status of HAI-1 and HAI-2 in a cell invasion progression model of lung cancer (CL1-0 and CL1-5). Highly invasive CL1-5 lung cancer cells were established and derived from the in situ lung adenocarcinoma cells (CL1-0) after 5-time isolations of invasive cells using Matrigel-coated transwell assays.^40^ We found that highly invasive CL1-5 cells expressed a low HAI-2 level compared to lowly invasive CL1-0 cells, while both cells lacked HAI-1 expression (Fig. 1b). The results together indicate that the expression levels of HAI-2 rather than HAI-1 are inversely correlated with the invasive and metastatic potentials of lung adenocarcinoma cells.

To investigate the correlation of HAI-2 expression levels with the clinical outcome, we utilised the Web resource, SurvExpress, to assess the prognostic significance of HAI-2 in lung adenocarcinoma (TCGA database, *n* = 255). The population of lung adenocarcinoma in the databank was divided into two groups: low risk and high risk, according to HAI-2 expression levels and Cox model.^41^ The result (Fig. 1c) showed that the high-risk group had a low level of HAI-2 expression (upper panel) with poor prognosis (lower panel) compared to the low-risk group. The data suggest that HAI-2 functions as a tumour suppressor for lung adenocarcinoma. We used immunohistochemical staining (IHC) to analyse HAI-2 protein levels in a lung adenocarcinoma tissue microarray, and classified the data into two groups: low and high expressions of HAI-2 (Fig. 1d). We found that the expression levels of HAI-2 were inversely correlated with high tumour invasion (T1/2 *v.s*. T3/4), cancer progression (stage I, II, III and IV) and early progression (stage I *v.s*. stage II, III, and IV) (Fig. 1e). These results together indicate that HAI-2 expression is down-regulated following the progression of lung cancer, and the low expression of HAI-2 is associated with poor prognosis.

### HAI-2 plays an inhibitory role in NSCLC cell migration and invasion

**Fig. 2:**
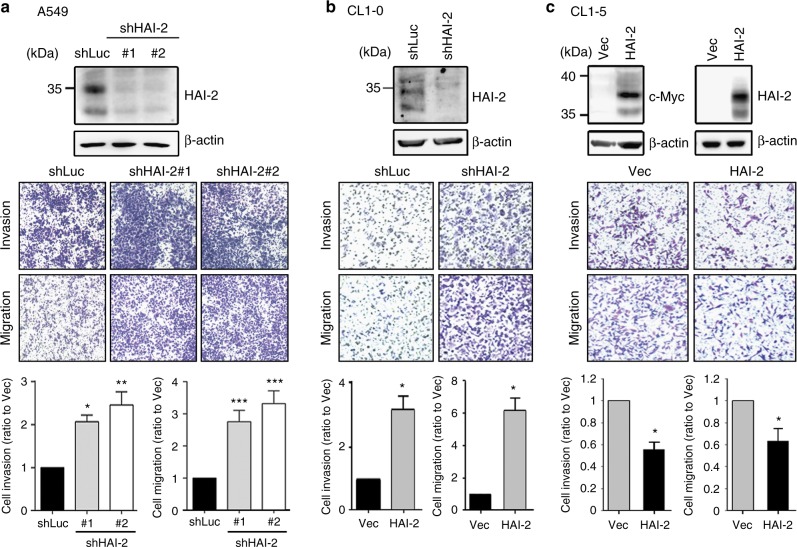
Role of HAI-2 in lung cancer cell motility. **a** Effect of HAI-2 knockdown on A549 lung cell migration and invasion. **b** Effect of HAI-2 silencing on CL1-0 cell migration and invasion. **c** Effect of HAI-2 overexpression on CL1-5 cell migration and invasion. CL1-5 cells were stably transfected with HAI-2.Myc construct for cell invasion and migration assays. The cells penetrating the transwells were counted and statistically calculated with a ratio to control cells (mean ± S.D., *n* = 3; *p < 0.05; **p < 0.005; ***p < 0.001). Magnification, x100

Since the gain of cell motility is a critical step to promote tumour invasive growth and metastasis,^42^ we investigated the role of HAI-2 in lung adenocarcinoma cell migration and invasion. The results from cell motility assays showed that HAI-2 silencing increased the cell migration and invasion of A549 cells (Fig. 2a). This effect can be rescued by re-expression of HAI-2 (Supplementary Figure S1). A similar result was also obtained from CL1-0 cells that HAI-2 knockdown significantly enhanced the cell migration and invasion (Fig. 2b). On the other hand, overexpression of HAI-2 suppressed the migrating and invasive abilities of highly invasive CL1-5 cells (Fig. 2c). The results together indicate that HAI-2 plays a suppression role in lung cancer cell motility.

### Identification of plasminogen as HAI-2’s target protease in NSCLC

**Fig. 3:**
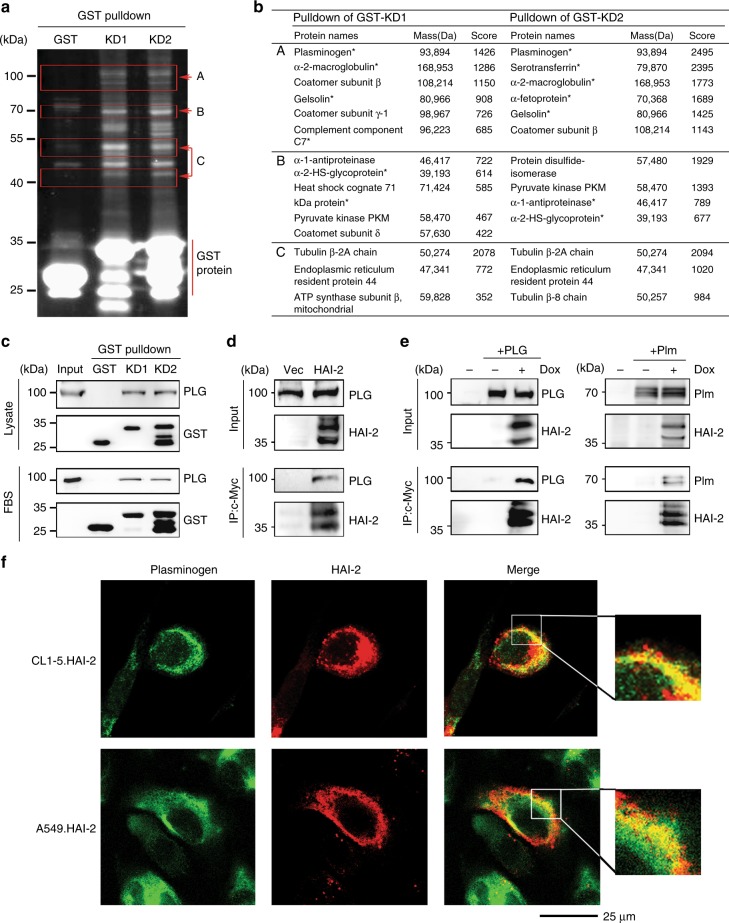
Identification of HAI-2’s associated proteins in lung cancer cells. **a** SYPRO Ruby-stained image of HAI-2’s associated proteins in an acrylamide gel after GST-pulldown and SDS-PAGE assays. The signals were captured by a UVP transilluminator. **b** The identity list of GST-pulldown proteins in (**a**) after LC-MS/MS analysis. **c** Immunoblot analyses of plasminogen after GST-HAI-2’s KD pulldown assays using CL1-5 cell lysate and FBS. **d** Co-immunoprecipitation of HAI-2 and plasminogen in HAI-2-overexpressing CL1-5 cells. HAI-2 proteins were pulled down from cell lysates by anti-c-Myc mAb (9E10). **e** Interaction of HAI-2 with plasminogen and plasmin using co-immunoprecipitation assays. A549 cells were transduced to carry a HAI-2.tet.aOn gene for HAI-2 overexpression upon doxycycline (Dox) induction. HAI-2 proteins were pulled down by anti-c-Myc mAb (9E10). The eluted samples were subjected to SDS-PAGE and immunoblotting using anti-PLG and anti-HAI-2 pAbs. **f** Subcellular localisation of plasminogen and HAI-2 using confocal microscopy. The cells were cultured on cell culture slides and immunocytochemically stained with anti-PLG pAb and anti-HAI-2 mAb (DC16), followed by secondary antibodies (Alexa Fluor 488 and 568) incubation. The images were captured by a confocal microscope

To elucidate the mechanism how HAI-2 inhibited lung adenocarcinoma cell motility, we set up to identify the target protease(s) of HAI-2 in lung adenocarcinoma cells. By using Q-PCR we first investigated if two well-known HAI-2’s target proteases (matriptase and hepsin^11,14^) were expressed in NSCLC. As shown in Supplementary Figure S2, there was none or little expression of matriptase in NSCLC A549, CL1-0, CL1-5 and H1299 cells. The expression levels of hepsin were low in highly invasive NSCLC CL1-5 and H1299 cells, compared to lowly invasive A549 and CL1-0 cells. Moreover, according to SurvExpress TCGA database, the results (Supplementary Figure S3) from the statistical analyses showed that a decreased expression level of matriptase was associated with a poor survival rate of lung cancer patients, while there was no association between hepsin expression levels and the patients’ survival. The data together suggest that matriptase or hepsin may not be the target of HAI-2 in NSCLC. Thus, we employed GST pulldown and LC/MS-MS analyses to isolate potential target(s) of HAI-2 in NSCLC. The fusion proteins of GST with the Kuntiz domain (KD) 1 and 2 of HAI-2 (GST-KD1 and GST-KD2) were expressed in CL1-5 cells. After GST pulldown, the isolated proteins were separated by SDS-PAGE with CYPRO-Ruby staining (Fig. 3a) and then cut out into three fragments (A, B and C), individually subjected to LC-MS/MS analyses. The identities of these proteins were listed in Fig. 3b. Among them, a serum-derived serine protease, plasminogen, received our attention because of its abundant presence in the pulldown products (Fig. 3b, row A). The data revealed that KD1 and KD2 domains of HAI-2 were able to interact with plasminogen from CL1-5 cell lysates as well as in fetal bovine serum (FBS) (Fig. 3c). The immunoprecipitation of HAI-2 from CL1-5 cell lysates also captured serum-derived plasminogen (Fig. 3d). A similar result (Fig. 3e) was observed that HAI-2 proteins were capable of interacting with both purified human plasminogen (PLG, left panel) and its active enzyme plasmin (Plm, right panel) in A549 cells. In addition, we illustrated the subcellular location of HAI-2 and plasminogen using immunofluorescence and confocal microscopy. The data showed that HAI-2 and plasminogen were co-localised on the cell surface of HAI-2-overexpressing CL1-5 and A549 cells (Fig. 3f). The results collectively indicate that plasmin(ogen) is one of HAI-2’s target proteins and both proteins can interact each other at the cell surface in lung adenocarcinoma cells.

### HAI-2 represses plasmin activity and plasmin-induced lung cancer cell motility

**Fig. 4:**
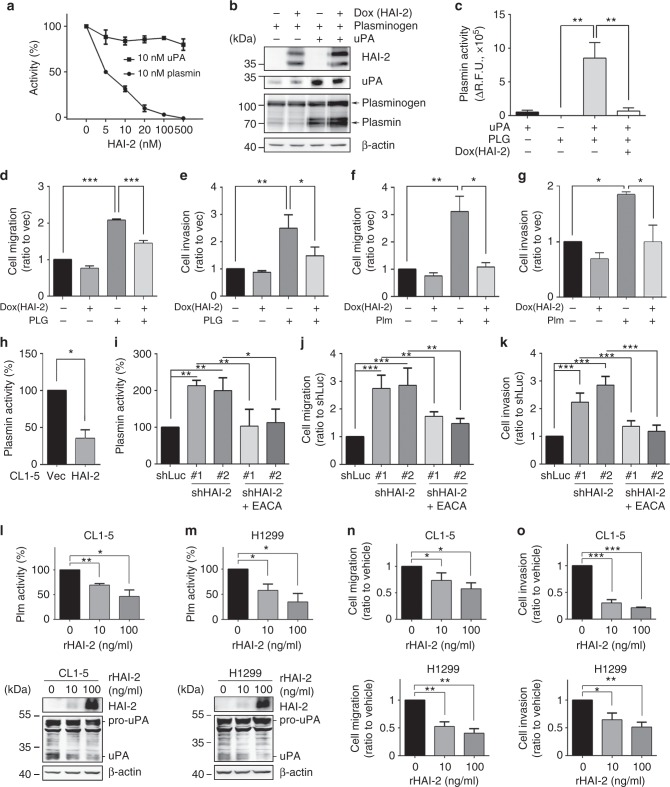
HAI-2 represses plasmin proteolytic activities and decreases lung cancer cell motility. **a** rHAI-2 inhibits the proteolytic activity of plasmin rather than uPA. Human active uPA and plasmin were incubated with rHAI-2 at the indicated concentrations. The artificial substrate (ALK-AMC for plasmin; GGR-AMC for uPA) were added to measure the protease activities (mean ± S.D., *n* = 3) **b** Examination of HAI-2 role in uPA-mediated plasminogen activation. After Dox induction for HAI-2 expression, cells were treated with or without 1 μg/ml human active uPA and 10 μg/ml human plasminogen. After 6 h, cell lysates were harvested for SDS-PAGE and immunoblotting. **c** Inhibitory effect of HAI-2 on the proteolytic activities of uPA-mediated plasminogen activation. After Dox induction for HAI-2 expression, 100 ng of human active uPA were added into cells and followed by adding 1 μg human plasminogen. The artificial substrate (ALK-AMC) was used to measure plasmin activities. (D/E) HAI-2 overexpression suppresses plasminogen-induced cell migration (**d**) and cell invasion (**e**). After serum starvation and EACA treatment, the cells were seeded to the transwells in the presence and absence of 10 μg/ml plasminogen (PLG) for cell migration and invasion assays. **f**/**g** HAI-2 overexpression suppresses plasmin-induced cell migration in the presence/absence of plasmin (Plm). **h** Analysis of cell-surface plasmin activity in HAI-2-overexpressing CL1-5 cells. **i** Analysis of cell-surface plasmin activity in HAI-2-knockdown A549 cells. The equal volume of 10 μM artificial substrate (ALK-AMC) was mixed with 100 μl of cell suspension to measure the cell-based plasmin activities. (mean ± S.D., *n* = 3; *p < 0.05). **j**/**k** Effect of plasmin inhibition on HAI-2 silencing-induced A549 cell migration (**j**) and cell invasion (**k**). **l**/**m** Effects of recombinant HAI-2 proteins on the cell-surface plasmin proteolytic activity in CL1-5 (**l**) and (**m**) H1299 lung cancer cells. **n**/**o** Effects of rHAI-2 on the migration (**n**) and invasion (**o**) of CL1-5 and H1299 cells. The cells passing through transwells were statistically calculated and represented as mean ± S.D. (*n* = 3; *p < 0.05; **p < 0.005; ***p < 0.001)

Since plasmin(ogen) has been shown to play a central role in cancer cell invasion,^16^ we addressed if HAI-2 could repress the proteolytic activity of plasmin or its activator uPA, leading to suppression of NSCLC cell invasion. We inspected the inhibitory effects of recombinant HAI-2 (rHAI-2) proteins on the proteolytic activities of plasmin and uPA. The rHAI-2 proteins were generated from insect cells and possesses simple glycosylations^43^ that give a single band in western blot.^39^ The results manifested that rHAI-2 proteins were capable of suppressing plasmin proteolytic activity but had no significant effect on uPA (Fig. 4a). Moreover, we found that HAI-2 expression could not repress uPA-induced activation of plasminogen by a proteolytic conversion into plasmin (Fig. 4b). To mimic the plasminogen activation system on the cell surface, we sequentially added purified human uPA and plasminogen into A549 cell cultures with or without HAI-2 overexpression. The results showed that the addition of uPA turned on the proteolytic activity of plasminogen, and HAI-2 was able to suppress the proteolytic activity of plasmin which was activated by uPA (Fig. 4c). The data together indicate that HAI-2 directly represses plasmin activity rather than uPA.

To examine if HAI-2-repressed cell migration and invasion were through inhibition of plasmin, we analysed the role of HAI-2 in plasmin(ogen)-induced A549 cell migration and invasion. The results showed that the addition of plasminogen or plasmin promoted the cell migration and invasion, and HAI-2 overexpression repressed plasminogen/plasmin-induced cell motility (Figs. 4d-g; Supplementary Figure S4). The added plasminogen was converted to plasmin by A549 cells in a time-kinetic manner and be up to approximately 97% activation of plasminogen to plasmin within 8 h. (Supplementary Figure S5). HAI-2 overexpression significantly reduced the cell-surface plasmin activity in CL1-5 cells (Fig. 4h), while HAI-2 silencing increased the proteolytic activity of cell-surface plasmin in A549 cells (Fig. 4i). A plasmin inhibitor, EACA (Epsilon-aminocaproic acid^44^), ably blocked HAI-2 knockdown-induced plasmin activity (Fig. 4i), the lung adenocarcinoma cell migration and invasion (Figs. 4j, k; Supplementary Figure S6). Since pro-uPA can be proteolytically activated by plasmin,^18^ HAI-2 overexpression reduced the levels of plasmin and active uPA in CL1-5 cells (Supplementary Figure S7A, left panel) and HAI-2 silencing increased the levels of plasmin and active uPA in A549 cells (Supplementary Figure S7A, right panel). Moreover, the treatment of EACA subsided cell-surface plasmin and decreased active uPA to basal levels (Supplementary Figure S7A, right panel). The results together indicate that HAI-2 inhibits cell-surface plasmin, leading to reducing active uPA and the reciprocal activation of plasminogen activation system (PAS). In addition, since metalloproteases-2 and 9 (MMP-2 and MMP-9) can be activated by plasmin,^21,45^ the data from zymography revealed a strong MMP-9 gelatinolytic activity in CL1-5 cells, and HAI-2 overexpression dramatically reduced the protease activity (~82 kDa, Supplementary Figure S7B, left panel). HAI-2 silencing increased the gelatinolytic activities of MMP-2 in A549 cells, and the treatment of EACA subsided the MMP-2 activity which was induced by HAI-2 silencing (~62 kDa, Supplementary Figure S7B, right panel). These results together indicate that HAI-2-repressed NSCLC cell motility, pro-uPA activation and MMP-2/9 activities are through suppressing cell-surface plasmin.

### Recombinant HAI-2 proteins repress cell-surface plasmin activities, NSCLC cell migration and invasion

Purified recombinant HAI-2 (rHAI-2) proteins containing the extracellular region of HAI-2 had been shown to repress prostate and breast cancer cell invasion.^7,39^ We assessed the effects of rHAI-2 proteins on the cell-surface plasmin, uPA and cell motility of highly invasive CL1-5 and H1299 NSCLC. The results showed that rHAI-2 proteins were able to inhibit the cell-surface plasmin activities and uPA activation of CL1-5 (Fig. 4l) and H1299 cells (Fig. 4m) in a dose-dependent manner. rHAI-2 also dramatically reduced the migratory (Fig. 4n) and invasive capabilities (Fig. 4o) of CL1-5 and H1299 cells (Supplementary Figure S8). The findings together indicate that rHAI-2 proteins with HAI-2’s extracellular region are capable of suppressing the cell-surface plasmin activities, uPA activation and NSCLC cell motility.

### HAI-2 inhibits an EMT-like transition of NSCLC through suppression of plasmin/uPA, HGF and TGFβ1 signalling

**Fig. 5:**
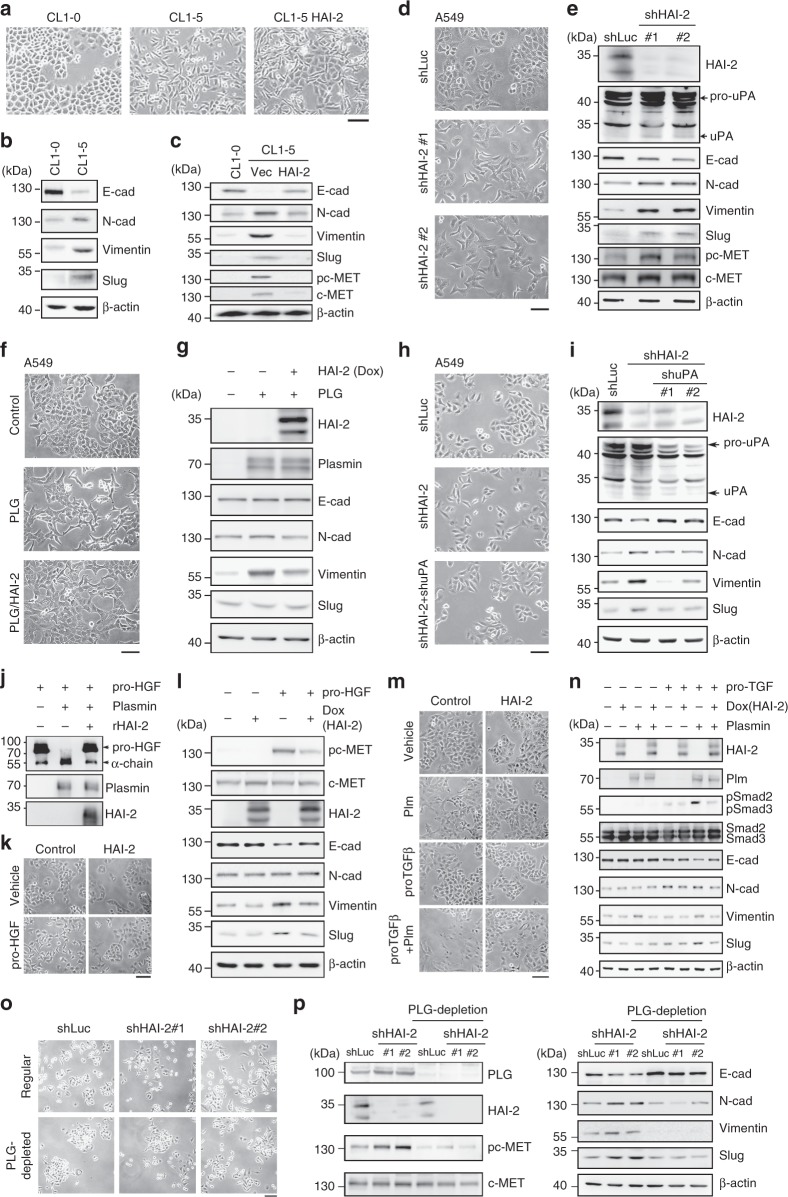
HAI-2-mediated mesenchymal epithelial transition of NSCLC *via* repressing plasmin activity, HGF/c-Met and TGFβ signalling. **a** The morphology of CL1-0, CL1-5, and HAI-2-overexpressing CL1-5 cells taken by a microscope. Scale bar = 100 µm. **b**/**c** Immunoblots of epithelial/mesenchymal cell biomarkers, phospho-c-Met and c-Met in CL1-0, CL1-5 and HAI-2-overexpressing CL1-5 cells. **d** The morphology of HAI-2 knockdown (shHAI-2 #1 and #2) and control (shLuc) A549 cells was pictured by a microscope. Scale bar = 100 µm. **e** Immunoblots of HAI-2, uPA, epithelial mesenchymal biomarkers, phospho-c-Met and c-Met in HAI-2 knockdown (shHAI-2 #1 and #2) and control (shLuc) A549 cells. **f** The morphology of A549 cells after the treatment of 10 µg/ml plasminogen or 1 µg/ml doxycycline in a serum-free culture condition for 48 h. Scale bar = 100 µm. **g** Immunoblots of HAI-2, plasmin, epithelial/mesenchymal biomarkers in A549 cells after the treatment. **h** The morphology of HAI-2 or uPA-knockdown A549 cells. Scale bar = 100 µm. **i** Immunoblots of HAI-2, uPA, and epithelial/mesenchymal biomarkers in HAI-2- or uPA-knockdown A549 cells. **j** Effect of plasmin and rHAI-2 on pro-HGF. 100 nM of pro-HGF, plasmin and rHAI-2 proteins were incubated in PBS for 2 h. Samples were then analysed by immunoblotting using anti-HGF (α-chain specific), anti-plasmin(ogen), and anti-HAI-2 pAbs. **k** The morphology of A549 cells after the treatment of 100 ng/ml pro-HGF or HAI-2 overexpression (Dox) for 48 h (scale bar = 100 µm). **l** Effect of pro-HGF and HAI-2 on the c-Met signalling and epithelial/mesenchymal markers. **m** The morphology of A549 cells after the treatment of plasmin, pro-TGFβ1 or HAI-2 overexpression (Dox) for 48 h. Scale bar = 100 µm. **n** Immunoblots of HAI-2, plasmin, Smad2/3 signalling, and epithelial/mesenchymal biomarkers in the presence or absence of pro-TGFβ1, HAI-2 or plasmin in A549 cells. **o** The morphology of HAI-2 knockdown (shHAI-2 #1 and #2) and control (shLuc) A549 cells in the culture media with normal or plasminogen-depleted FBS (scale bar = 100 nm). **p** Immunoblot analyses of plasminogen, HAI-2, c-Met signalling, and epithelial/mesenchymal biomarkers in HAI-2 knockdown (shHAI-2 #1 and #2) and control (shLuc) A549 cells with regular or plasminogen-depleted FBS

The highly invasive CL1-5 cells exhibit a low level of E-cadherin compared to their primary, lowly invasive CL1-0 cells.^46^ We also observed that CL1-5 cells had a spindle-like phenotype compared to epithelial-like CL1-0 cells, and HAI-2 overexpression restored the phenotype of CL1-5 cells to CL1-0 cells (Fig. 5a). The inspection of epithelial/mesenchymal markers showed that CL1-5 cells had an up-regulation of N-cadherin and Vimentin (two mesenchymal cell markers), and a decreased level of E-cadherin (an epithelial biomarker), indicating that CL1-5 cells exhibit a mesenchymal phenotype (Fig. 5b). Interestingly, HAI-2 overexpression restored the expression of E-cadherin and down-regulated N-cadherin and Vimentin, suggesting that HAI-2 can promote the mesenchymal epithelial transition (MET) of lung cancer cells (Fig. 5c). We analysed the gene expression levels of four EMT-related transcription factors (Slug, Snail, Twist and ZEB2) using Q-PCR in CL1-5, CL1-0 and HAI-2-knockdown A549 cells. The data showed that only Slug dramatically increased in CL1-5 cells compared to CL1-0 cells (Supplementary Figure S9A) and HAI-2 silencing enhanced Slug expression in A549 cells (Supplementary Figure S9B). Interestingly, HAI-2 overexpression pushed the transition of CL1-5 cells from mesenchymal to epithelial characteristics (an increase of E-cadherin and decreased levels of N-cadherin, Vimentin and Slug) (Fig. 5c). Since the HGF/c-Met signal pathway has been reported to mediate EMT,^47^ the protein and phosphorylation levels of c-Met were up-regulated in CL1-5 cells and down-regulated after HAI-2 overexpression in the cells (Fig. 5c, lower panel). HAI-2 silencing also promoted EMT-like transitions of A549 cells, including morphological alterations, cell scattering (Fig. 5d), down-regulation of E-cadherin, activation of uPA, and up-regulation of N-cadherin, Vimentin, Slug and c-Met signalling (Fig. 5e). The results together suggest that HAI-2 is a regulator of the MET of NSCLC.

To investigate whether plasmin(ogen) was involved in HAI-2-mediated MET of NSCLC, A549 cells were treated with or without plasminogen in the presence or absence of doxycycline for HAI-2 overexpression. The addition of plasminogen promoted the morphological changed to a spindle-like phenotype with increasing cell protrusions (Fig. 5f), while HAI-2 expression attenuated the effect of plasminogen on the biological events of NSCLC (Fig. 5g). The conversion of plasminogen to plasmin occurred after its addition into cell cultures (Figs. 4b and 5g). The administration of plasminogen dramatically up-regulated Vimentin while had less effects on E-cadherin, N-cadherin or Slug (Fig. 5g). HAI-2 overexpression subsided the levels of plasminogen-increased Vimentin (Fig. 5g). These results together indicate that HAI-2 can repress plasminogen/plasmin-induced morphological alterations and Vimentin expression in NSCLC.

Since knockdown of HAI-2 increased the levels of uPA and the EMT of NSCLC, we examined whether uPA played a role in promoting the EMT of NSCLC. shRNA approaches were employed to knock down HAI-2 or uPA in A549 cells. The results (Fig. 5h) showed that HAI-2 knockdown promoted the cell scattering, and uPA silencing attenuated the degree of cell scattering in HAI-2-knockdown A549 cells. Similar to the results in Fig. 5e, HAI-2 knockdown decreased the protein levels of E-cadherin and up-regulated N-cadherin, Vimentin, and Slug in A549 cells (Fig. 5i). Silencing of uPA was able to revert the HAI-2 knockdown-induced changes on E-cadherin, N-cadherin, Vimentin and Slug in the cells (Fig. 5i). The results together indicate that uPA plays an important role in the EMT of NSCLC when HAI-2 is down-regulated.

Since PAS plays a critical role in hepatocyte growth factor (HGF) activation^48^ that promotes the EMT of NSCLC,^49^ we examined if HGF/c-Met signalling was involved in HAI-2-mediated MET of NSCLC. The data of the in vitro assay showed that active plasmin was able to proteolytically cleave pro-HGF (indicated by a release of the α-chain of HGF), and recombinant HAI-2 (rHAI-2) proteins ably inhibited plasmin function for the proteolytic cleavage of pro-HGF to HGF (Fig. 5j). In the presence of pro-HGF under a serum-free condition for 48 h, the morphology of A549 cells was elongated and overexpression of HAI-2 suppressed pro-HGF-induced morphological alteration of A549 cells (Fig. 5k). c-Met signalling was highly induced by the addition of pro-HGF into the cells, and the HGF-induced c-Met signalling was attenuated upon HAI-2 overexpression (Fig. 5l). HGF-induced c-Met signalling down-regulated E-cadherin and up-regulated Vimentin and Slug, but did not considerably change N-cadherin; all of HGF/c-Met-altered events were reverted after HAI-2 overexpression (Fig. 5l). The results together indicate that HAI-2 promotes the MET of NSCLC in part *via* suppressing the plasmin-mediated proteolysis of pro-HGF and c-Met signalling.

In addition to HGF, TGF-β1 has been shown to be another potent inducer for the EMT of cancers.^50,51^ Since it has been documented that plasmin is involved in TGF-β1 activation,^24,52^ we investigated whether the HAI-2-plasmin axis could modulate TGF-β1 activation for the EMT of NSCLC. To assess the role of the HAI-2-plasmin axis in TGF-β1 activation of NSCLC, the morphology of A549 cells and TGF-β signalling were examined after the treatment of pro-TGF-β1 or plasmin. As shown in Fig. 5m, pro-TGF-β1 alone did not significantly change the morphology of A549 cells. The co-treatment of pro-TGF-β1 and plasmin transformed A549 epithelial cells into a spindle-like, mesenchymal phenotype. HAI-2 overexpression attenuated plasmin-TGF-β1-induced morphological change of NSCLC (Fig. 5m). Activation of pro-TGF-β1 by plasmin was indicated by the increased phosphorylation levels of Smad2, a downstream effector of the TGF-β signal pathway (Fig. 5n). HAI-2 overexpression ably subsided the role of plasmin in the activation of pro-TGF-β1 (Fig. 5n). Similarly, the co-treatment of plasmin and pro-TGF-β1 dramatically decreased E-cadherin and up-regulated Vimentin and Slug, while the HAI-2 overexpression suppressed the effects of plasmin and pro-TGF-β1 on the above biological events (Fig. 5n). The plasminogen depletion reduced the induction effects of HAI-2 knockdown on NSCLC A549 cell scattering (Fig. 5o). The EMT characteristics in HAI-2-silencing NSCLC were reverted after plasminogen was depleted (Fig. 5p). The results together suggest that plasmin is an important protease in HAI-2-silencing-induced EMT of NSCLC, and indicate that HAI-2 plays an inhibitory role in plasmin-induced activation of pro-HGF and pro-TGF-β1 for the EMT of NSCLC.

### Knockdown of HAI-2 promoted lung cancer metastasis and EMT in a xenografted mouse model

**Fig. 6:**
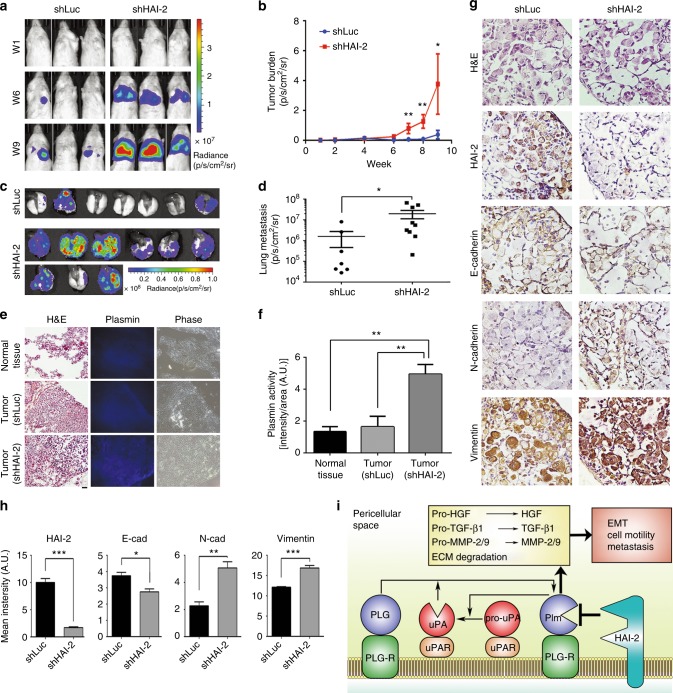
Down-regulation of HAI-2 increases a lung metastatic ability of A549 cells, the tissue plasmin activity and EMT. **a** HAI-2-knockdown (shHAI-2) and control (shLuc) A549 cells were intravenously injected into the tail-veins of NOD/SCID mice (1 × 10^6^ cells per mouse). The metastatic cells in mouse lungs were tracked by bioluminescence and measured by in vivo imaging system (Xenogen IVIS 50, Perkin Elmer). The images from three representative mice in each experimental group were shown in week 1, 6 and 9 after tumour injection. **b** The intensities of bioluminescence of metastatic cells were quantified by Living Image Software and statistically calculated by GraphPad Prism 6 (mean ± SEM., shLuc, *n* = 7; shHAI-2, *n* = 9; *p < 0.05; **p < 0.005. Student’s *t*-test). **c** Metastatic lesions in mouse lungs. Mice were scarified in week 10 after tumour injection and the lungs were harvested. The bioluminescence of metastatic cells was visualised by in vivo imaging system. **d** Quantification of metastatic lesions in mouse lungs. The bioluminescence of metastatic cells in mouse lungs was quantified and statistically calculated (mean ± SEM., shLuc, *n* = 7; shHAI-2, *n* = 9; *p < 0.05; **p < 0.005. Student’s *t*-test). **e** In situ plasmin proteolytic activity in mouse lungs. The frozen sections of the mouse lung tissues covered with artificial substrates (ALK-AMC) and incubated at 37 °C for an hour. The in situ plasmin activities were visualised by a fluorescent microscopy using UV excitation (360 nm). H&E: hematoxylin and eosin stain; Plasmin: *in situ* zymography of plasmin; Phase: phase contrast images. Scare bar = 100 μm. **f** Quantification of *in situ* plasmin proteolytic activity in normal and tumour tissues (mean ± SD, *n* = 3; **p < 0.01; one-way ANOVA). **g** Immunohistochemical staining of HAI-2, E-cadherin, N-cadherin and Vimentin in the frozen sections of mouse lung tissues. Scare bar = 100 μm. **h** Quantification of the IHC intensity in four different tumour lesions of each experiment. The intensity of the IHC images was measured and quantified by ImageJ. **i** The model of HAI-2-repressing uPAS, EMT, cell motility and metastasis of lung adenocarcinoma

To assess the roles of HAI-2 in NSCLC metastasis, we intravenously injected HAI-2-knockdown A549 cells and control cells into immunodeficient mice and analysed tumour formation in the mouse lungs. The result showed that HAI-2-knockdown induced A549 cell colonisation in the lungs after tail vein injection, compared to control cells, indicating that down-regulation of HAI-2 increased the lung metastatic ability of NSCLC (Fig. 6a-d). Moreover, the tumours with HAI-2 knockdown possessed higher plasmin activities than control tumours and normal tissues (Fig. 6e, f). To investigate whether the down-regulation of HAI-2 could promote the EMT of the lung cancer cells in metastatic lesions, we analysed the epithelial and mesenchymal markers in the sections of lung metastatic tumours. The data revealed that the tumour lesions with HAI-2 knockdown exhibited a mesenchymal phenotype (a decreased level of E-cadherin and increased levels of N-cadherin and Vimentin), compared to control (Fig. 6g, h). These findings together indicate that down-regulation of HAI-2 increases the EMT of NSCLC and metastasis, at least in part *via* an increase of plasmin activities.

## Discussion

Dysregulation of pericellular proteolysis has been implicated in cancer progression and metastasis. In this study, we found that a membrane-anchored serine protease inhibitor-HAI-2 was down-regulated in highly invasive NSCLC. We identified plasmin to be a target protease of HAI-2 in NSCLC. A novel molecular mechanism was shown that HAI-2 serves as a novel non-covalent inhibitor of plasmin and suppresses the protease-induced activations of pro-HGF, pro-TGF-β1 and MMP-2/9 as well as ECM degradation, leading to reducing lung cancer cell motility, EMT, and metastasis (Fig. 6i). The findings indicate that the down-regulation of HAI-2 during the NSCLC progression promotes cell invasion and metastasis *via* an elevation of plasmin activity, activations of pro-HGF and pro-TGF-β1, and promotion of EMT.

Given that HAI-2 has a broadly inhibitory spectra against pericellular proteases,^8^ down-regulation of HAI-2 may result in the aberrant activations of cell-surface protease cascades, culminating in a proteolytic storm and tumour cell dissemination. For example, HAI-2 can suppress prostate cancer cell invasion, tumour growth and metastasis *via* targeting a membrane-anchored serine protease matriptase.^11^ However, NSCLC showed none or few expressions of matriptase (Supplementary Figure S2 & S10). The other target of HAI-2, hepsin, also promotes cancer progression.^14,53^ Unexpectedly, the expression levels of hepsin were decreased in the highly invasive lung adenocarcinoma (CL1-5 and H1299) (Supplementary Figure S2), compared to lowly invasive A549 and CL1-0 cells. The analyses of TCGA database revealed that matiptase is down-regulated in the high-risk group with a low survival rate in lung adenocarcinoma patients (Supplementary Figure S3A), while hepsin levels is not relevant with the lung cancer patients’ survival (both low-risk and high-risk groups have a similar survival rate, Supplementary Figure S3B). In addition, matriptase briefly expresses in two of five lung cancer cell lines,^54^ and hepsin is amplified only in 5.4% cases of lung adenocarcinoma.^53^ The results together suggest that matriptase and hepsin may not be implicated in HAI-2 downregulation-mediated lung adenocarcinoma progression. Instead, down-regulation of HAI-2 in lung adenocarcinoma could promote cell invasion and metastasis *via* an increase of plasmin activity. The data together indicate that HAI-2 exhibits a distinct regulation on its target serine protease in different cancers; e.g., the HAI-2/matriptase axis for prostate cancer^11,39^ versus the HAI-2/plasmin axis for NSCLC. The exact pair between HAI-2 and its specific target in the other cancers requires more investigations.

The uPA and uPA receptor (uPAR) are expressed in lung adenocarcinoma cells (Supplementary Figure S11 and ref. ^27^). Moreover, two SERPIN-type inhibitors, PAI-1 and α-2-antiplasmin, have been well-studied and irreversibly inhibit uPA and plasmin activity, respectively.^55^ α-2-antiplasmin has been identified only to repress free plasmin but unable to inhibit cell-surface plasmin (Supplementary Figure S12 and ref. ^56^). Interestingly, HAI-2 is newly identified to be a membrane-anchored and non-covalent inhibitor to modulate the PAS system by directly inhibiting cell-surface plasmin, leading to suppression of the PAS system. The inhibition of HAI-2 on plasmin shows a distinct enzyme-inhibitor kinetic from α-2-antiplasmin on plasmin or PAI-1 on uPA (Supplementary Figure S13). In the presence of equimolar uPA and plasminogen, the low (1:10) and high ratios (1:1) of PAI-1 to uPA have a similar inhibitory activity on plasmin (~60% inhibitions), while HAI-2 possess a dose-dependent manner to inhibit plasmin activity [~75% inhibition at a high ratio (10:1) of HAI-2 to uPA/plasminogen and ~20% inhibition at a low ratio (1:1)] (Supplementary Figure S13A). For inhibiting plasmin, α-2-antiplasmin shows a more effective suppression on plasmin (~40% at a low ratio to plasmin, and over 99% at a high ratio), while the high ratio of HAI-2 to plasmin exhibited up to 66% inhibition (Supplementary Figure S13B). Thus, the results imply that HAI-2 has a less inhibitory effect on plasmin than α-2-antiplasmin. In NSCLC, α-2-antiplasmin has no function on cell-surface plasmin, while membrane-anchored HAI-2 serves as an important inhibitor for cell-surface plasmin to suppress the PAS system. Without HAI-2’s inhibition, cell-surface plasmin becomes constitutively active for massive ECM degradation and growth factor activation, leading to invasive lung tumour growth and metastasis.

The decreased expression of HAI-2 promoted an EMT-like process in lung cancer cells. Our data manifest that uPA is required for the EMT of lung cancer cells which is induced by HAI-2 down-regulation. Activation of two pro-growth factors (pro-HGF and pro-TGF-β1) is regulated by the plasmin-HAI-2 axis. On the other hand, it had no significant effect on EGFR signalling because the administration of plasmin could not affect EGFR in the NSCLC (Supplementary Figure S14). Our clinical results showed that HAI-2 was significantly down-regulated in the lung cancer patients with high tumour invasion (T3 + T4 *v.s*. T1 + T2). Since EMT plays an important role in the tumour dissemination,^28^ the down-regulation of HAI-2 may be a factor to promote tumour dissemination in lung adenocarcinoma *via* up-regulation of plasmin and the PAS. Therefore, the expression levels of HAI-2 in tumour lesions may serve as a prognostic marker to evaluate the metastatic risk of lung adenocarcinoma.

In Figs. 5e, c, the alteration folds of the EMT markers in HAI-2-silencing A549 cells were not as high as the effects of HAI-2 overexpression on those markers in CL1-5 cells. The discrepancy of EMT marker changes after the alteration of HAI-2 expression in CL1-5 and A549 cells may be due to the nature of cell lines, including the different levels of uPA between both cells (Supplementary Figure S11). Since CL1-5 cells have a higher level of uPA (Supplementary Figure S11) and plasminogen activation than A549 cells (20% plasmin for CL1-5 versus 1% plasmin for A549, under a regular culture condition, Supplementary Figure S15), CL1-5 cells would be more sensitive to the alteration of HAI-2 than A549 cells. Thus, HAI-2 overexpression can efficiently repress plasmin and c-Met activity in CL1-5 cells. In A549 cells, HAI-2 silencing can increase the EMT with a less degree than that of the increased MET in CL1-5 cells after HAI-2 overexpression, because of the low plasminogen activation (~1% plasmin) in A549 cells compared to CL1-5 cells (~20% plasmin, Supplementary Figure S15).

Although the previous reports have shown that only Kunitz domain 1 of HAI-2 is responsible for inhibiting HGFA, matriptase and prostate cancer cell invasion,^39,57^ we find that both Kunitz domains of HAI-2 can suppress plasmin activities (Supplementary Figure S16A and S16B) and cell motility (Supplementary Figure S16C and S16D) in NSCLC. Because most of NSCLC have no or a trace of matriptase expression (Supplementary Figure S2 and S10), HAI-2-mediated NSCLC cell motility and metastasis may be mainly through regulating plasmin rather than matriptase.

The cell-surface receptors for uPA and plasminogen are crucial for the activation of the PAS. In general, pro-uPA binds to uPA receptor (uPAR) for a proteolytic conversion to uPA by the cell-surface plasmin.^58^ In comparison with uPAR, the plasminogen receptors are miscellaneous in various cell types;^59^ Among them, cell-surface Cytokeratin 8 and 18 (CK8/18) have been identified to be a plasminogen receptor in cancer.^60^ Our data revealed that CK8/18 physically interacted with plasminogen and HAI-2 in NSCLC (Supplementary Figure S17A~S17C), and co-localised at the cell surface (Supplementary Figure S17D). The pericellular locations of HAI-2 and plasminogen were also confirmed by the co-localisation with E-cadherin (Supplementary Figure S18&S19). The data suggest that CK8/18 may function as a plasminogen receptor and cooperate with HAI-2 to modulate the PAS system in NSCLC.

In summary, this study shows a novel mechanism that HAI-2 regulates the cell-surface plasmin and functions as a potent inhibitor for the PAS system of NSCLC. The down-regulation of HAI-2 is one of the causes to promote EMT, cell invasion and metastasis. In addition, the purified recombinant HAI-2 proteins exhibit an inhibitory activity against plasmin and NSCLC invasion, suggesting a therapeutic potential of HAI-2 in targeting plasmin and suppression of NSCLC metastasis.

## Supplementary information

Supplementary Figures, Methods and legends
