# Supplementary material for: HAI-2 as a novel inhibitor of plasmin represses lung cancer cell invasion and metastasis
Source: Br J Cancer. 2019 Feb 15;120(5):499–511. doi: 10.1038/s41416-019-0400-2 (PMC6461989; doi:10.1038/s41416-019-0400-2)
Supplement: Supplementary file 1 — Supplementary Figures, Methods and legends [file 41416_2019_400_MOESM1_ESM.docx]

**Supplementary Figures**

**Supplementary Figure S1**


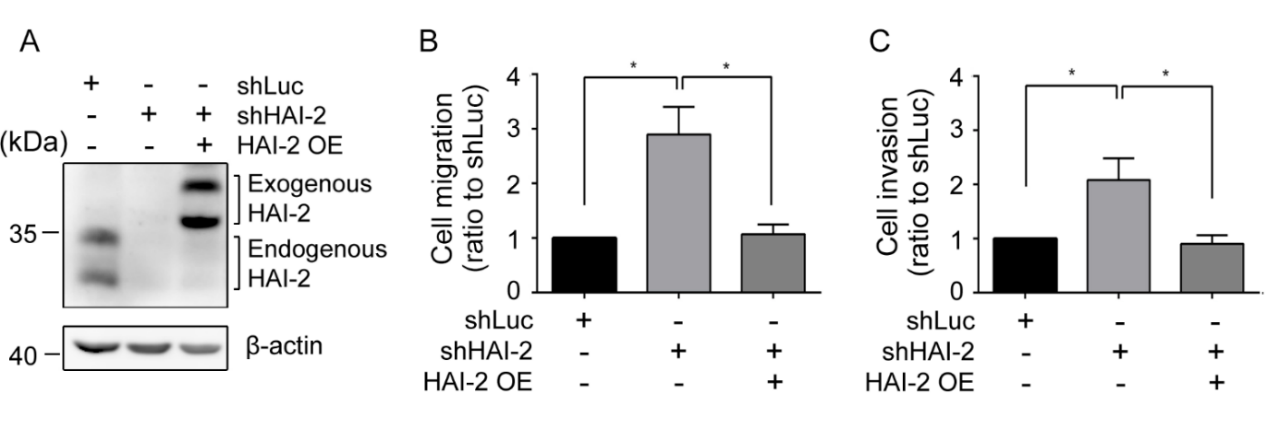


**Supplementary Figure S1. Re-expression of HAI-2 reduced the HAI-2-silencing-induced cell migration and invasion of NSCLC cells.** (A) Immunoblot analysis of HAI-2 in A549 control cells (shLuc), HAI-2-knockdown cells (shHAI-2, #1:TRC0000073578) and HAI-2-knockdown/overexpression cells [shHAI-2 and HAI-2 overexpression (OE)]. Cells were cultured in regular media for 3 days and then harvested for SDS-PAGE and western blot analysis using an anti-HAI-2 antibody. The overexpressed HAI-2 (exogenous) possesses a higher molecular weight than the endogenous HAI-2 because of the extra C-terminal tags and cloning site regions. β-actin was used as an internal control. (B/C) Examination of the cell migration (B) and invasion (C) of A549 control cells (shLuc), HAI-2-knockdown cells (shHAI-2, #1:TRC0000073578) and HAI-2-knockdown/overexpression cells [(shHAI-2 and HAI-2 (OE)] using transwell assays. For cell migration and invasion assays, transwells were coated without or with Matrigel (BD biosciences), respectively. Cells were seeded at a density of 5x10^4^ cells per upper well of transwells. Regular culture media (10% FBS) were added at the bottom wells as a chemoattractant. After seeding, cells were then incubated for 24 hours. The cells penetrating to the bottom surfaces of transwells were stained with 1 % crystal violet, imaged and statistically quantified by Image J from three independent experiments (mean±SD, n=3; *, *p*<0.05; one-way ANOVA).

**Supplementary Figure S2**

**
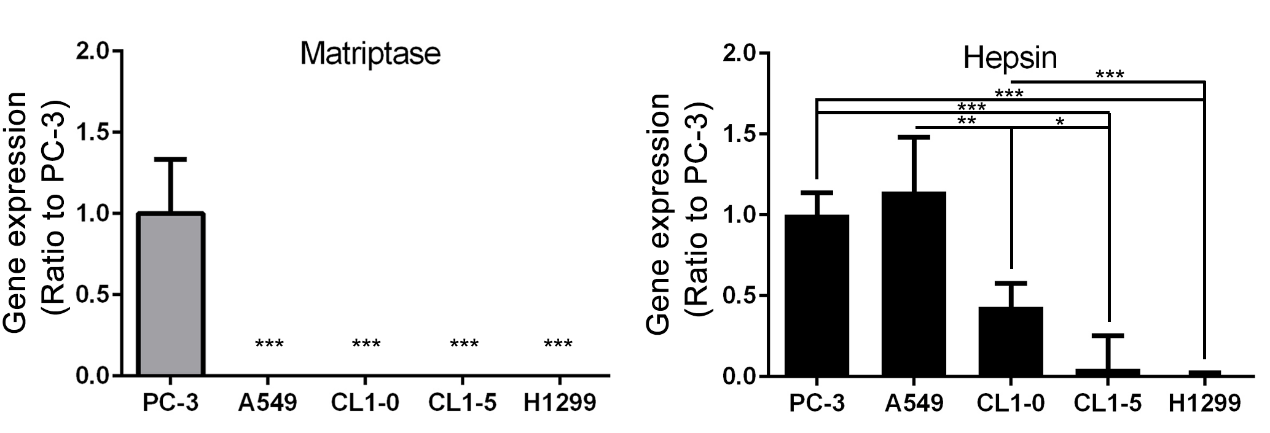
**

**Supplementary Figure S2. Gene expression levels of matriptase and hespin in lung adenocarcinoma cell lines.** The gene expression levels of matriptase and hepsin were analyzed by real-time RT-PCR with normalization to GAPDH. The relative gene expression levels in different SNCLC cells were further normalized to those in prostate cancer PC-3 cells which served as a positive control. (mean±SD, n=3; *, *p*<0.05; **, *p*<0.01; ***, *p*<0.001; One-way ANOVA)

**Supplementary Figure S3**

**
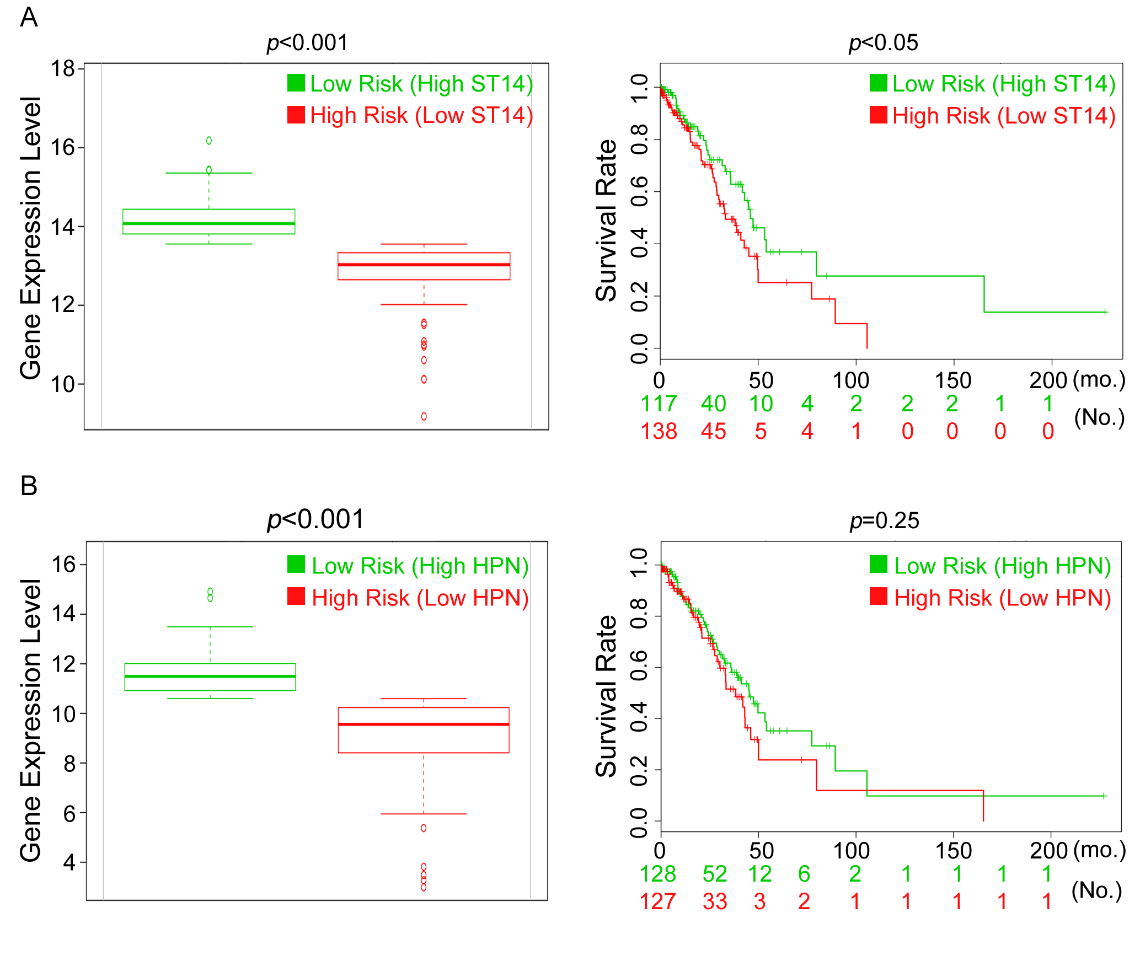
**

**Supplementary Figure S3. Association of matriptase (ST14) and hepsin (HPN) expressions with survival rates of lung adenocarcinoma patients.** (A) The samples of 255 lung adenocarcinoma patients (SurvExpress, #13 TCGA database) were divided into High risk and Row risk groups according to the matriptase expression levels (ST14, left panel). The survival rates of patients in the High risk and Low risk groups were analyzed and shown in the right panel. (B) The samples (SurvExpress, #13 TCGA database) were divided into the High risk and Row risk groups according to the hespsin expression levels (HPN, left panel). The survival rates of patients in the High risk and Low risk groups were analyzed and shown in the right panel. The results showed that a decreased expression level of matriptase is associated with a low survival rate of lung cancer patients, while there is no association between hepsin expression levels and lung cancer patients’ survival.

**Supplementary Figure S4**


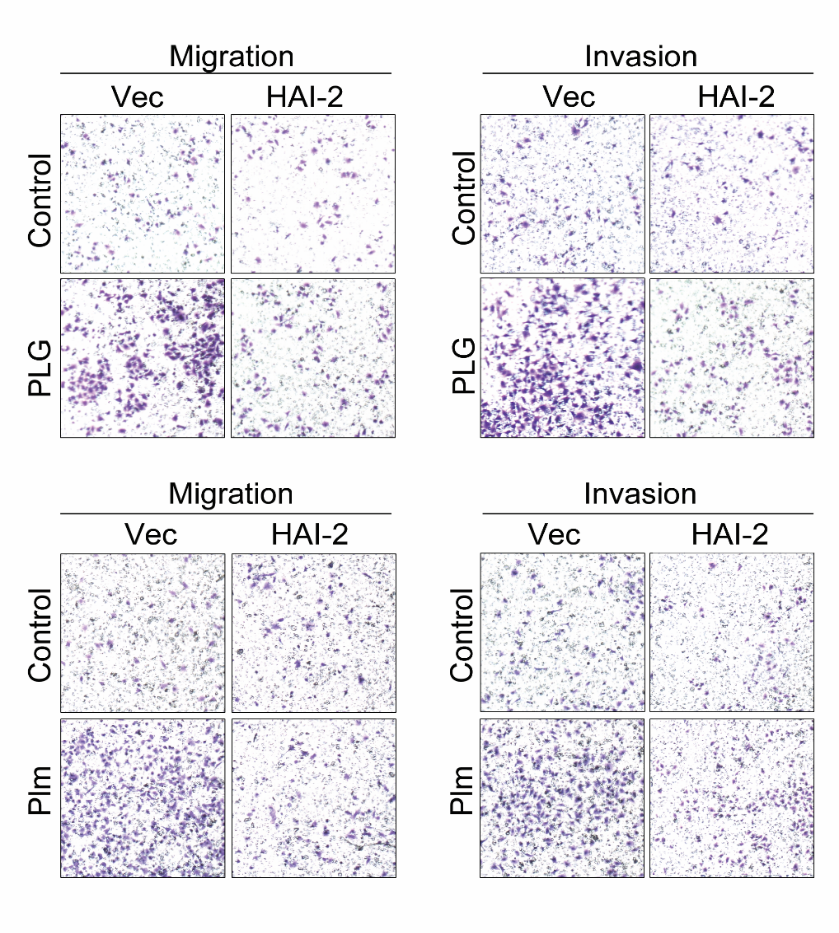


**Supplementary Figure S4. HAI-2 represses plasmino(gen)-induced NSCLC cell migration and invasion.** After serum starvation and EACA treatment, A549 cells were treated with Doxycycline (1 μg/ml) for HAI-2 overexpression. Control cells were treated with vehicle. Cells were then seeded at a density of 5x10^4^ cells per transwell coated with or without 1 μg Matrigel in the presence/absence of 10 μg/ml plasminogen (PLG) or plasmin (Plm) for cell invasion (40 h) and migration assays (24 h), respectively. The cells traveling through transwells were stained with crystal violet. The images of migratory and invasive cells on the bottoms of the transwells were photographed using a CCD camera under a microscope (magnification, x100). Three independent experiments were performed. A set of representative images from the cell migration and invasion assays were shown.

**Supplementary Figure S5**





**Supplementary Figure S5**. **Conversion of plasminogen into plasmin at 0.5, 2 and 8 hours after an addition of plasminogen into A549 cell cultures.** A549 cells were seeded at a density of 5x10^5^ cells per well of 6-well dishes. After 24 hours, cells were refreshed with serum-free medium and incubated for 16 hours. Purified human plasminogen proteins (10 μg/ml) were then added to the cell cultures and incubated for 0.5, 2 and 8 hours. Cell lysates were then harvested for western blot analyses using an anti-plasminogen antibody. β-actin was used as an internal control.

**Supplementary Figure S6**
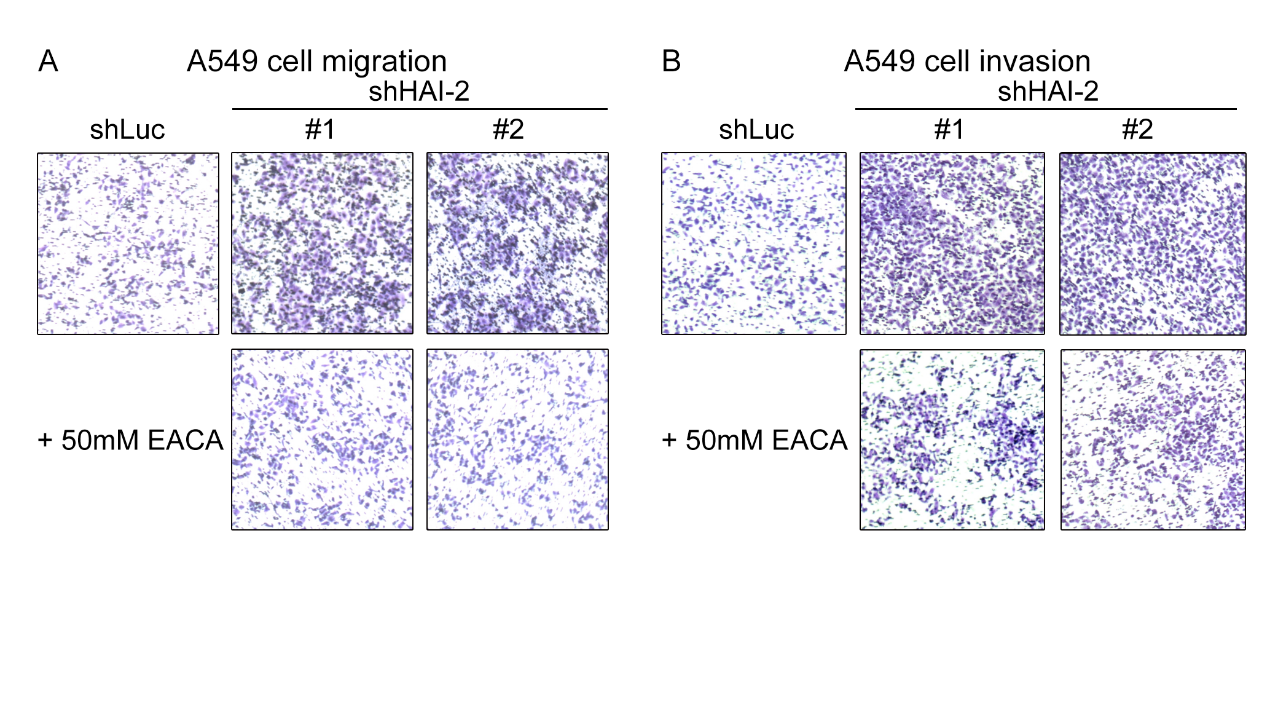


**Supplementary Figure S6. Effect of plasmin inhibitor EACA on HAI-2-knockdown-increased A549 cell migration (A) and invasion (B).** Serum-starved HAI-2-knockdown A549 and control shLuc A549 cells were seeded at a density of 5x10^4^ cells per transwell coated with or without 1 μg Matrigel in the presence or absence of 50 mM EACA for cell invasion (40 h) and migration assays (24 h), respectively. The cells passing through transwells were stained with crystal violet. The images of migratory and invasive cells on the bottoms of transwells were imaged using a CCD camera under a microscope (magnification, x100). Three independent experiments were performed. A set of representative images from the cell migration (A) and invasion assays (B) were shown.

**Supplementary Figure S7**


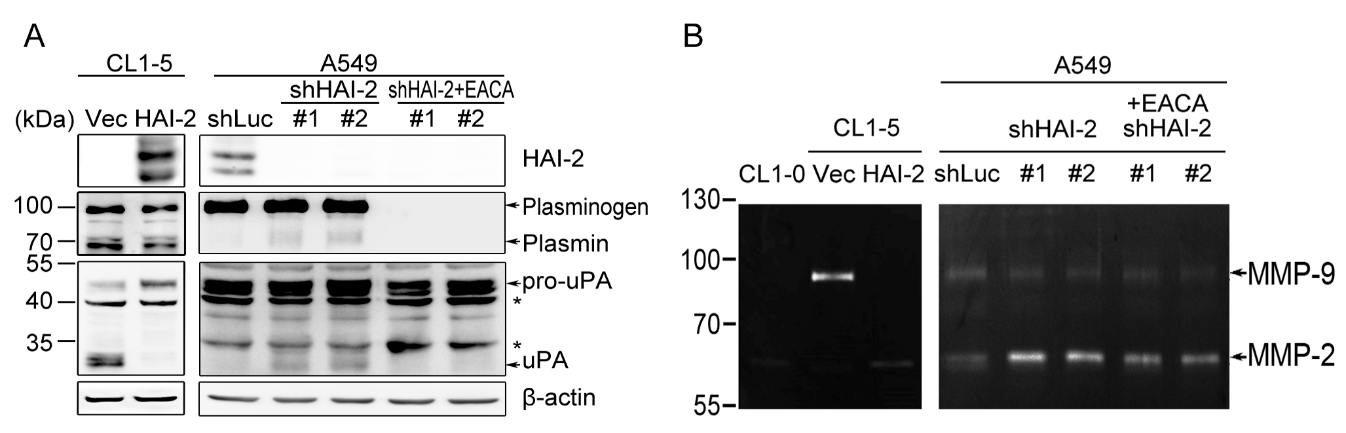


**Supplementary Figure S7. HAI-2 represses uPA and MMP-2/9 activation.** (A) HAI-2-down-regulated uPA activation. HAI-2-knockdown A549 cells were treated with or without 50 mM EACA overnight. Cells (HAI-2-overexpressing and Vec CL1-5 cells; HAI-2-knockdown and shLuc A549 cells) were then collected, lyzed and subjected to SDS-PAGE and immunoblot analyses using anti-HAI-2 pAb and anti-uPA pAb. The cell-surface plasminogen and plasmin were eluded from intact cells by 50 mM EACA and detected by anti-PLG pAb. β-actin was used as control. (B) Gelatinolytic zymography in the conditioned media of HAI-2-overexpressing CL1-5 and HAI-2-knockdown A549 cells. Cells were seeded at a density of 5x10^5^ cells per well in 6-well dishes. Next day the media were refreshed with OPTI-MEM. shHAI-2 A549 cells were treated with or without 50 mM EACA. Cells were then incubated overnight and their conditioned media were subjected to SDS-PAGE within 0.1% gelatin gels and zymographic assays with Coomassie Brilliant Blue R-250 staining.

**Supplementary Figure S8**


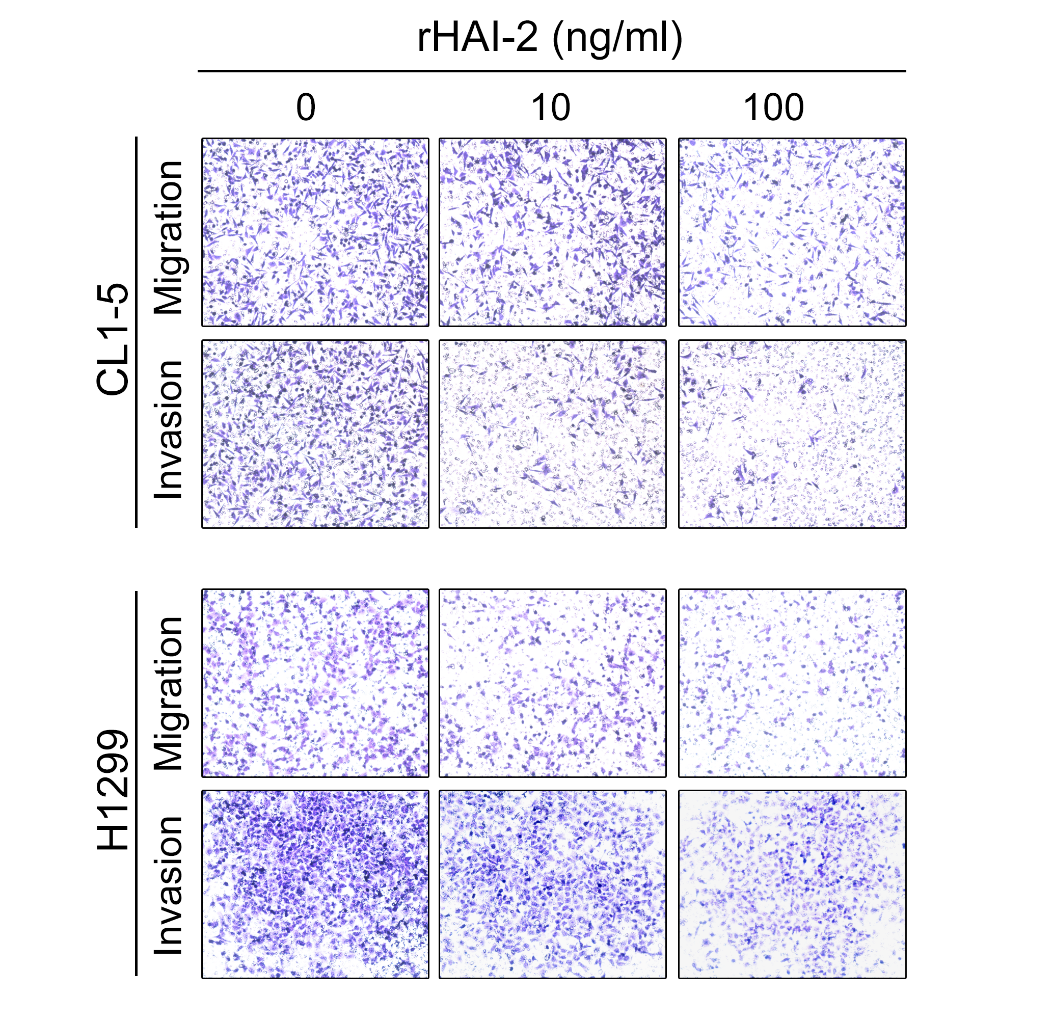


**Supplementary Figure S8. Recombinant HAI-2 represses NSCLC cell migration and invasion. NSCLC** CL1-5 and H1299 cells were seeded at a density of 2x10^4^ per transwell. The culture media were added with the indicated concentrations of recombinant HAI-2 proteins. For cell migration and invasion assays, the transwells were evenly coated with or without 1 μg Matrigel and cells were incubated in the transwells for 40 and 24 hours for cell invasion and migration assays, respectively. The cells passing through transwells were stained with crystal violet. The images of migratory and invasive cells on the bottoms of transwells were photographed using a CCD camera under a microscope (magnification, x100). Three independent experiments were performed. A set of representative images from the cell migration and invasion assays were shown.

**Supplementary Figure S9**



**Supplementary Figure S9. Examination of the expression levels of E-cadherin transcriptional repressors (Slug, Snail, Twist and ZEB2) in CL1-0 and CL1-5 cells (A) and HAI-2-knockdown A549 cells (B) using Q-PCR.** RNA was purified using TRIzol reagent following the manufacturer’s protocol. cDNA was synthesized by cDNA synthesis kit using 5 ng RNA, according to the manufacturer’s protocol. The gene expression levels of Slug, Snail, Twist and ZEB2 were measured by Q-PCR (StepOne real-time PCR system, Thermo Fisher, MA, USA) using SYBR green and specific primer pairs (see below). The expression levels of these genes were normalized to internal control GAPDH, statistically calculated with normalization to the control groups, and represented as means±S.D. Each assay was performed in three independent experiments (*, *p*<0.05, Student’s t-test).

| Slug | 5’-GCCTCCAAAAAGCCAAACTAC-3’  5’-GTGTGCTACACAGCAGCAGCC-3’ |
| --- | --- |
| Snail | 5’-GCTCCTTCGTCCTTCTCCTCTA-3’  5’-GGCACTGGTACTTCTTGACA-3’ |
| Twist | 5’-TGTCCGCGTCCCACTAGC-3’  5’-TGTCCATTTTCTCCTTCTCTGGA-3’ |
| ZEB2 | 5’-GCGGCATATGGTGACACACAA-3’  5’-CATTTGAACTTGCGATTACCTGC-3’ |
| GAPDH | 5’-AAAGGATCCACTGGCGTCTTCACCACC-3’  5’-GAATTCGTCATGGATGACCTTGGCCAG-3’ |

**Supplementary Figure S10**

**
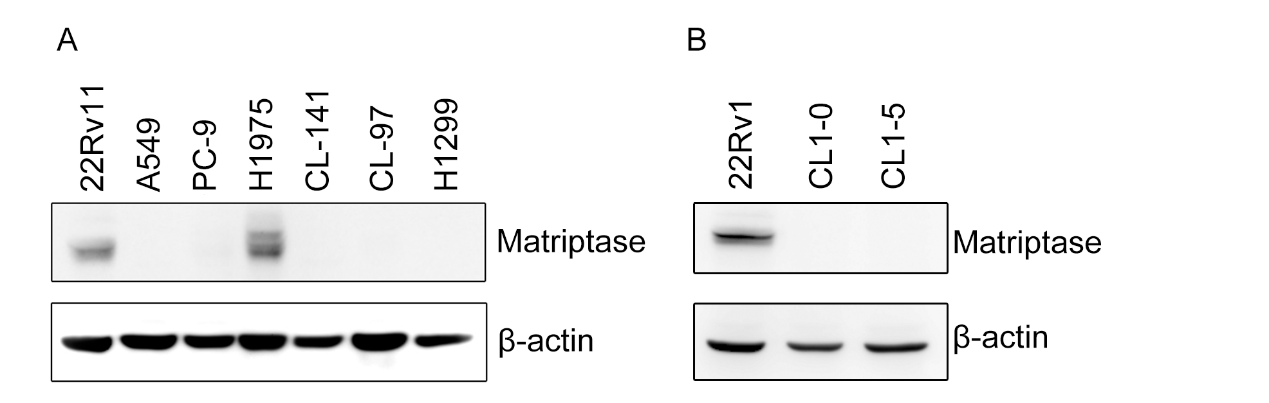
**

**Supplementary Figure S10. Matriptase expression levels in different lung adenocarcinoma cancer cells.** To examine the protein levels of matriptase in different lung adenocarcinoma cells, prostate cancer CWR22Rv1 cells were used as a positive control. Cell lysates were taken from different lung adenocarcinoma cells and subjected to SDS-PAGE and immunoblot analyses using an anti-total matriptase mAb (M32) (a gift from Dr. Chen-Yong Lin at the Georgetown University, Washington, DC, USA^1^). β-actin was used as control.

**Supplementary Figure S11**


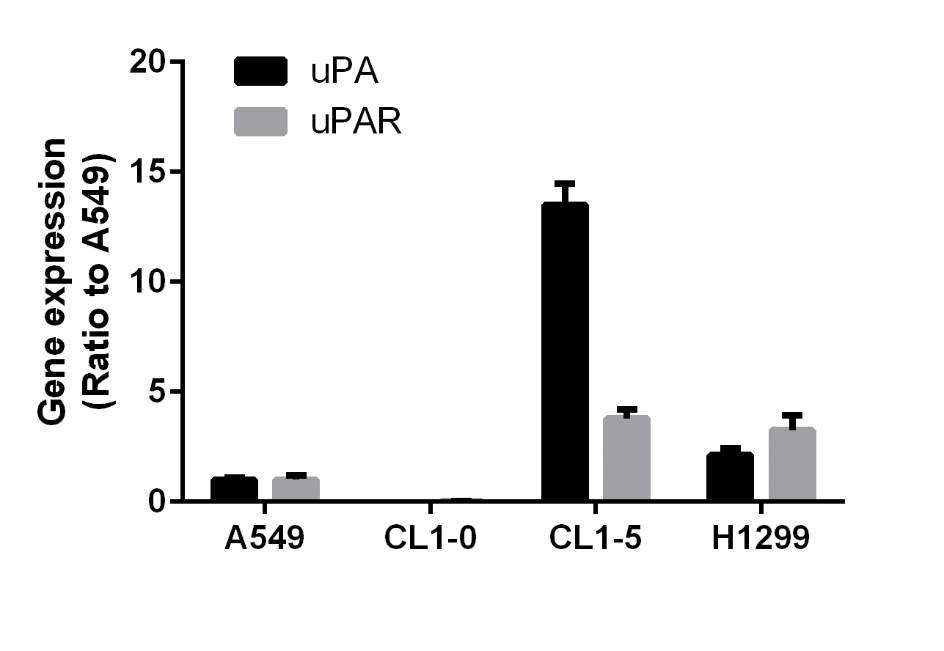


**Supplementary Figure S11. Gene expression levels of uPA and uPAR in lung adenocarcinoma A549, CL1-0, CL1-5 and H1299 cells.** The gene expression levels of uPA and uPAR in lung adenocarcinoma A549, CL1-0, CL1-5 and H1299 cells were analyzed using real-time RT-PCR with normalization to GAPDH. The gene expression levels were further statistically normalized to those in A549 cells.

**Supplementary Figure S12**


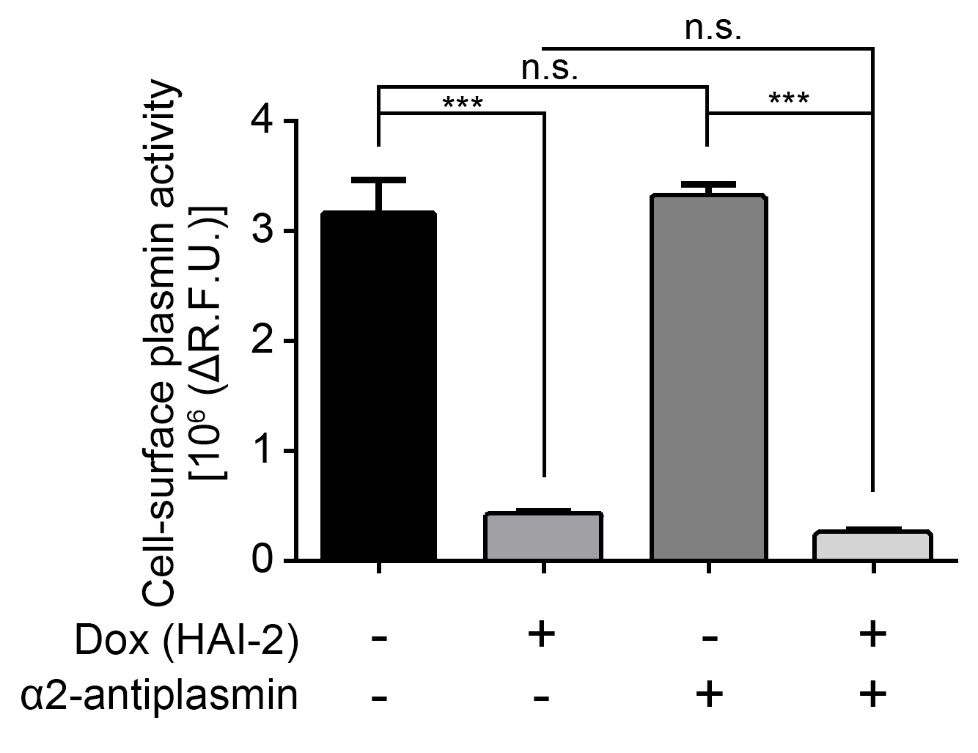


**Supplementary Figure S12. α2-antiplasmin is unable to repress cell-surface plasmin activity.** HAI-2-inducible A549 cells (HAI-2.aOn cells) were treated with 1 μg/ml doxycycline (Dox) overnight for inducing HAI-2 expression. Control cells were treated with vehicle. Ten nanomolars (nM) of human plasmin (Abcam) were added to each well for 2 hours at room temperature (RT). One hundred nM of human α2-antiplasmin (Abcam) were added to cells for 30 min at RT. After two washes, the cells were detached by citric saline and 2x10^4^ cells were mixed with 10 μM ALK-AMC for the plasmin activity assay. The increased fluorescence (EX:360nm/EM:465nm) generated from the cleavage of substrates after 30 min at 37°C was detected by a microplate reader (Paradigm, Beckman Coulter, CA, USA) and statistically calculated from three independent experiments. (***, *p*<001; n.s., *p*>0.05; one-way ANOVA)

**Supplementary Figure S13**


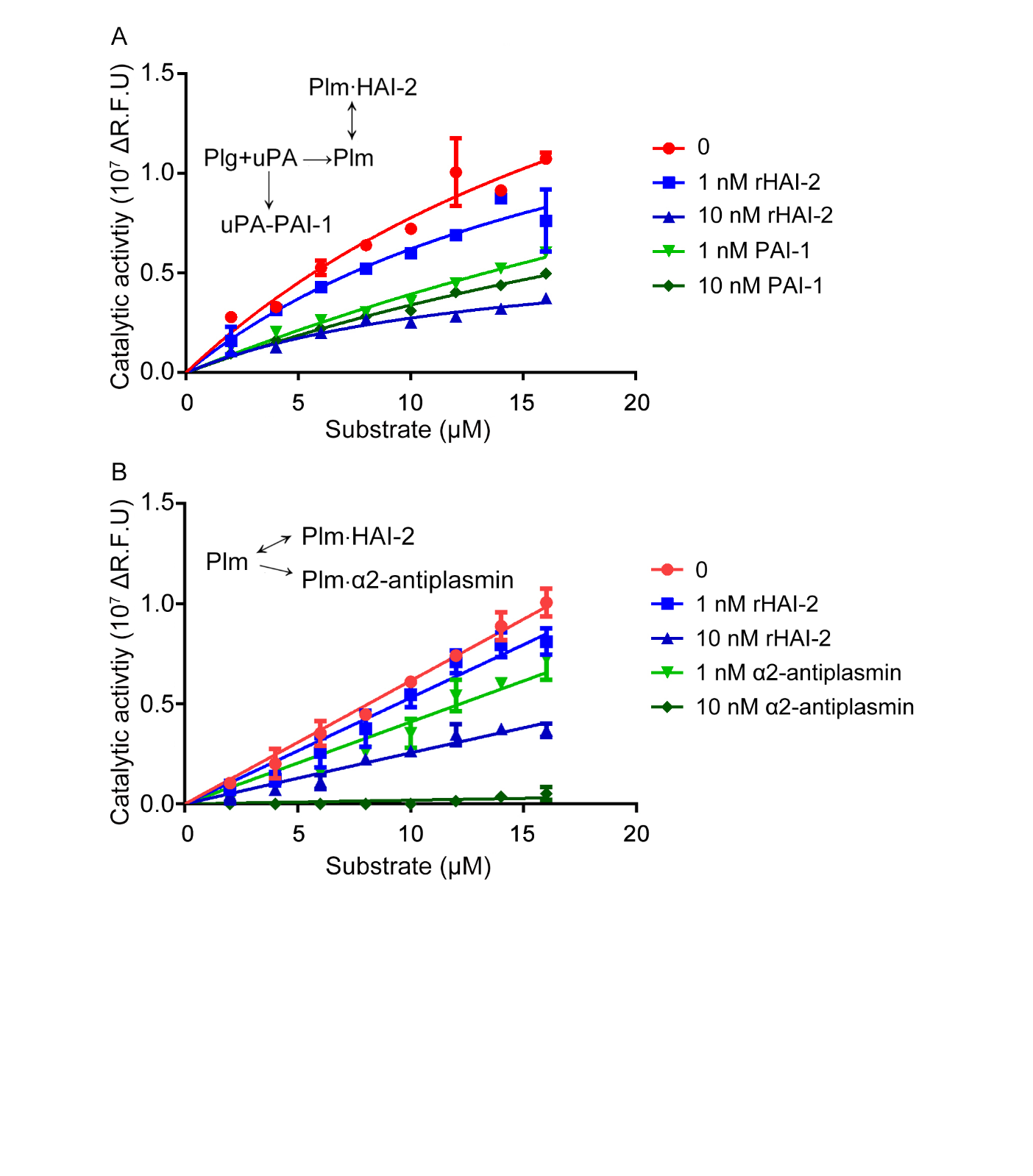


**Supplementary Figure S13. The enzyme-inhibitor kinetics of HAI-2, PAI-1, and α-2-antiplasmin in the plasminogen activation system.** (A) Analyses of the roles of HAI-2 and PAI-1 in the plasminogen activation system. To examine the roles of HAI-2 and PAI-1 in inhibiting the plasminogen activation system, 10 nM plasminogen (Abcam, UK) and 10 nM uPA (Merck Millipore, Germany) were mixed with the indicated concentrations of rHAI-2 (purified from baculovirus expression system) or rPAI-1 (Abcam, UK) in PBS. After incubation at RT for 30 min, the indicated concentrations of the substrates (ALK-AMC, Sigma-Aldrich, MO, USA) were added to the reaction solutions. The fluorescent intensity (EX: 360 nm; EM: 465 nm) (ΔR.F.U.) after a 30-min reaction was measured using a microplate reader (SpectraMax Paradigm, Molecular Device, CA, USA) and statistically calculated from three independent experiments. (B) Examination of the effects of purified rHAI-2 and α-2-antiplasmin on the proteolytic activity of plasmin. For the enzymatic assay, 10 nM plasmin (Abcam, UK) were mixed with the indicated concentrations of purified rHAI-2 or α-2-antiplasmin (Abcam, UK) in a reaction buffer (50 mM Tris, pH 7.4). After incubation at RT for 30 min, the indicated concentrations of the substrates (ALK-AMC) were added to the reaction solution. The fluorescent intensity (ΔR.F.U., EX: 360 nm; EM: 465 nm) after a 30-min reaction was measured by a microplate reader and statistically calculated from three independent experiments.

**Supplementary Figure S14**

**
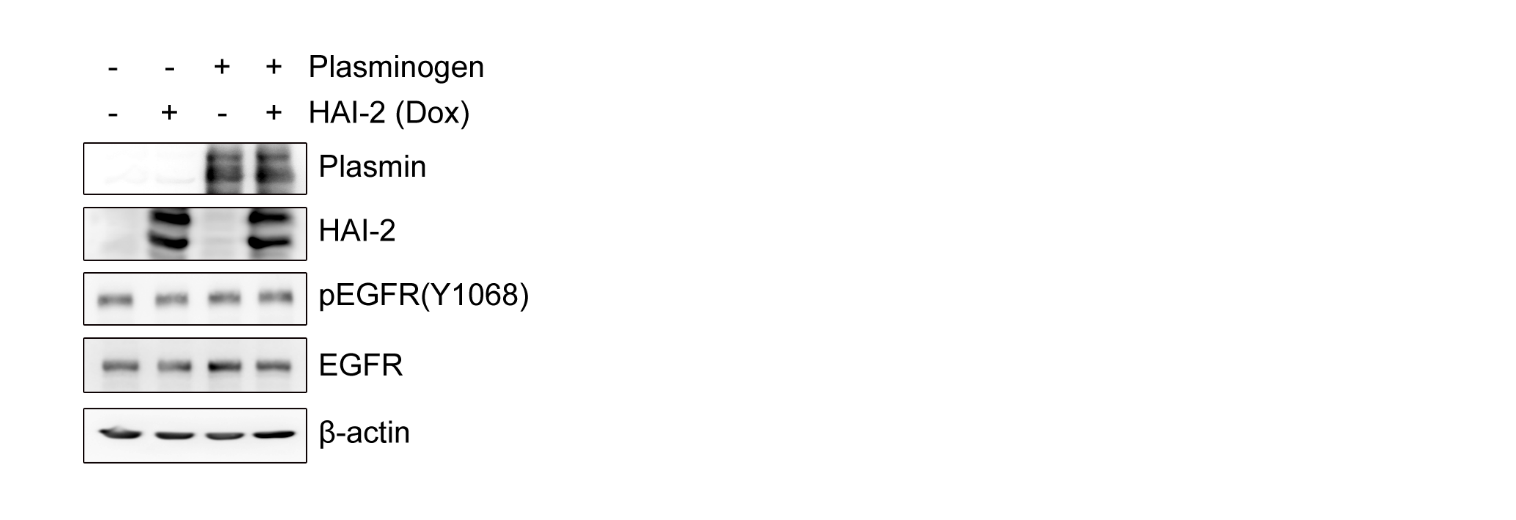
**

**Supplementary Figure S14. Analyses of the roles of plasmin and HAI-2 in EGFR signaling of NSCLC cells.** A549 HAI-2.aOn cells were treated with 1 μg/ml doxycycline to induce HAI-2 expression. Control cells were treated with vehicle alone. Next day the media were refreshed with OPTI-MEM in the presence or absence of 10 μg/ml plasmin, and incubated for 16 hours. Cell lysates were then subjected to SDS-PAGE and immunoblotting using anti-plasmin, anti-HAI-2, anti-EGFR (Merck Millipore, Germany) and anti-phospho-EGFR (Y1068, Cell signaling, MA, USA) Abs. β-actin was used as control.

**Supplementary Figure S15**

**

**

**Supplementary Figure S15. Levels of plasminogen and plasmin in A549 an CL1-5 cells.** (A) Immunoblots of plasminogen and plasmin in A549 and CL1-5 cells. Cells were cultured under a regular condition for 2 days and underwent 50 mM EACA extraction to release the cell-surface plasminogen and plasmin. The eluted solutions were collected for western blot analyses using an anti-plasminogen pAb (GeneTex). (B) Analysis of the percentages of plasminogen and plasmin in the plasminogen system in A549 and CL1-5 cells. The quantification was performed using Image J according to the images in (A).

**Supplementary Figure S16**

**
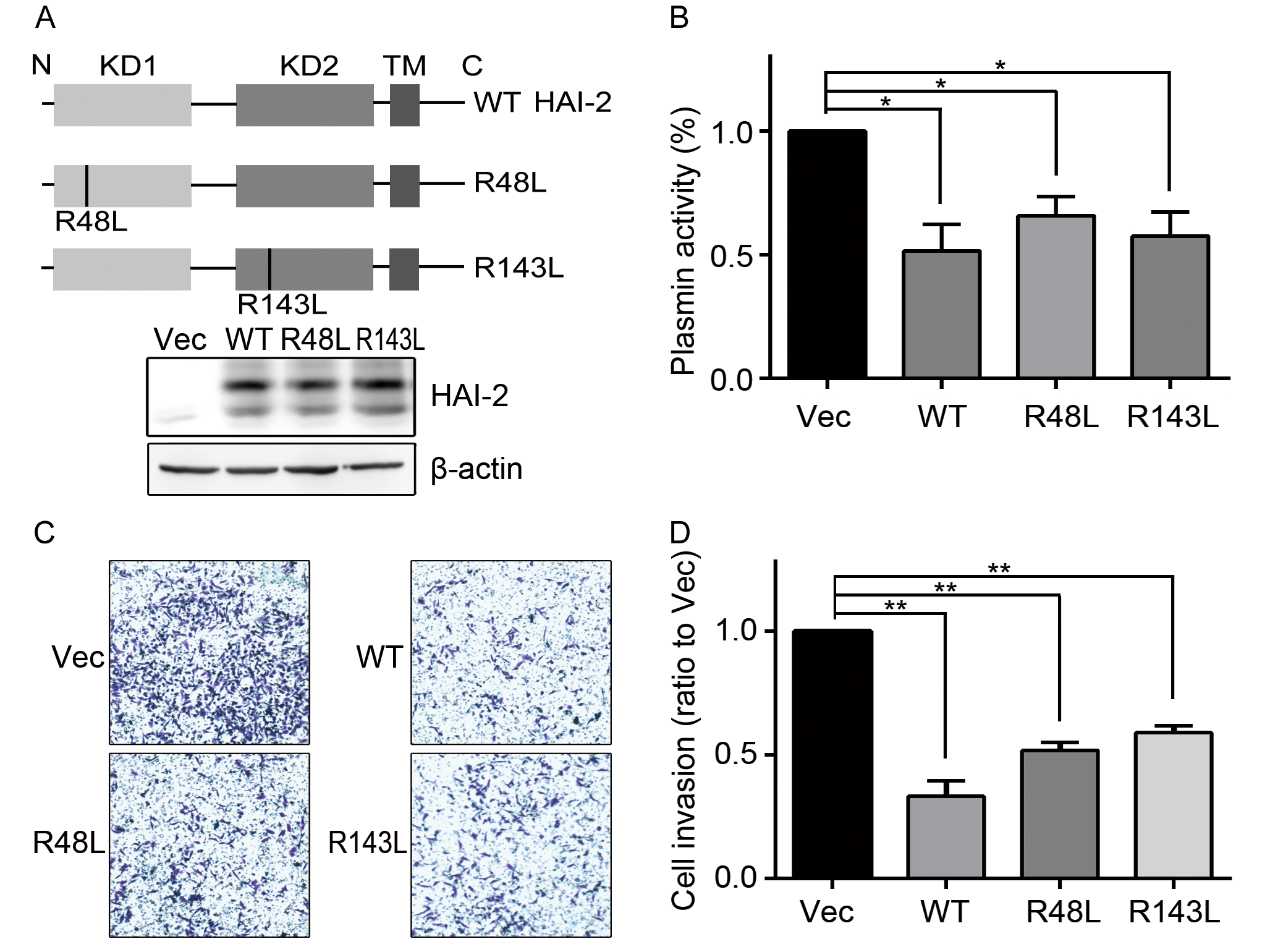
**

**Supplementary Figure S16. Analyses of different HAI-2 mutant proteins on the proteolytic activity of cell-surface plasmin and the NSCLC cell invasion.** (A) Schematic structures of wild-type and mutant HAI-2 constructs with Kunitz domain mutations (R48L for KD1; R143L for KD2, upper panel). These constructs were cloned into pCDNA3.1-Myc-His vectors. The plasmids were transfected into CL1-5 cells using Lipofectamine. Control cells were transfected with vectors. Two days later, stable pools were selected by 500 μg/ml G418 for 2 weeks. The exogenous HAI-2 protein levels in the cells were detected using immunoblotting with an anti-Myc mAb (9E10, Santa Cruz, CA, USA). β-actin was used as control. The results were shown in the bottom panel. (B) Analyses of different HAI-2 mutant proteins on the proteolytic activity of cell-surface plasmin in CL1-5 cells. CL1-5 cells were detached from the bottom of culture dishes and suspended in PBS with a concentration of 200 cells per microliter. For the proteolytic activity assays of cell-surface plasmin, 100 μl of cell suspension (2x10^4^ cells) were taken and mixed well with 100 μl of 10 μM artificial substrate (ALK-AMC). Then the plasmin activity was revealed by the fluorescence (ΔR.F.U.; EX: 360 nm; EM: 465 nm) which was generated in a 30-minute reaction period and recorded by a microplate reader. The results were performed in three independent experiments, statistically calculated and represented as mean±S.D. (n=3). *; *p*<0.05; One-way ANOVA. (C/D) Examination of the roles of different HAI-2 mutants in NSCLC cell invasion. Serum-starved CL1-5 cells were seeded at a density of 5x10^4^ cells per transwell coated with 1 μg Matrigel for cell invasion assays (16 h). The cells traveling through transwells were stained with 1 %crystal violet. The invasion assays were performed in three independent experiments. One set of representative images were shown in (C). Cell numbers were counted, statistically calculated and represented as mean±S.D. (n=3). (D) **; *p*<0.005; One-way ANOVA.

**Supplementary Figure S17**

**
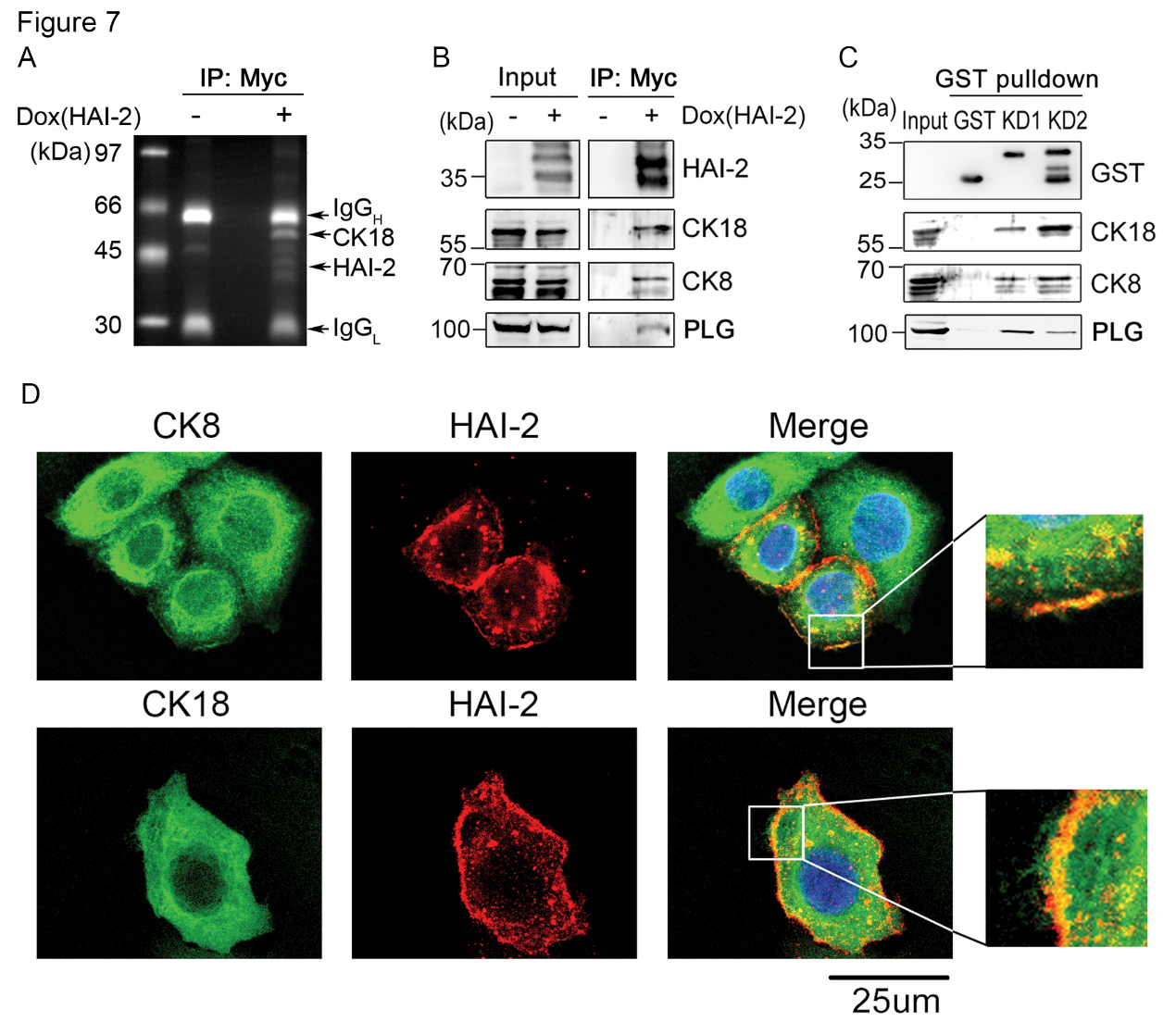
**

**Figure S17. Cytokeratin 8/18 (CK8/18) is associated with HAI-2 and plasminogen.** (A) Co-immunoprecipitation of HAI-2-Myc with Cytokeratin 18. Thirty milligrams of HAI-2-overexpressing A549 cell lysates were incubated with 20 μg of anti-Myc mAb at 4°C overnight. The pulldown products were eluted with pH 2.5 glycine buffer and separated with SDS-PAGE. SYPRO Ruby staining revealed a ~60-kDa protein precipitated with HAI-2, which was identified as Cytokeratin 18 (CK18) by LC/MS-MS. (B) Following the same experimental procedures, the co-immunoprecipitated products of HAI-2 were analyzed using immunoblotting with anti-HAI-2, anti-CK8, anti-CK18 and anti-PLG (plasminogen) pAbs. (C) One milligram of A549 cell lysates was mixed with 3 μg of GST-fusion proteins (GST control, GST-KD1 and GST-KD2) and then incubated at 4°C for 1 hour. The associated proteins were isolated by glutathione sepharose beads and analyzed by immunoblotting using anti-CK8, anti-CK18 and anti-PLG pAbs. (D) Subcellular localization analysis of CK8/18 and HAI-2 in A549 cells using confocal microscopy. HAI-2-overexpressing A549 cells were cultured on a cover slide (Cat. 30108, SPL life science, Korea) for 24 hours. After fixation with 4% paraformaldehyde, cells were blocked with 5% horse serum and then incubated with anti-CK8, anti-CK18 and anti-HAI-2 (DC16) Abs at 4°C overnight. After three washes with PBS, The Alexa Fluor secondary antibodies (anti-rabbit Ab labeled with Alexa Fluor 488; anti-mouse Ab labeled with Alexa Fluor 568, Thermo Fisher, MA, USA) were applied to the slides. The images were captured by a confocal microscope (TCS SP5, Leica, Germany). Scale bar= 25 μm.

**Supplementary Figure S18**

**
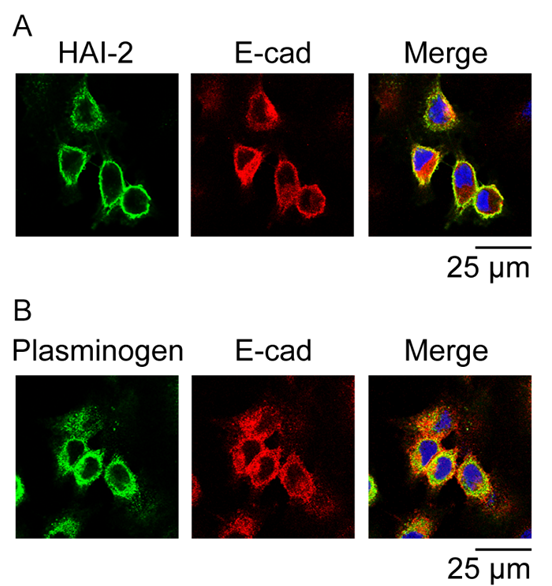
**

**Supplementary Figure S18. Subcellular localization of HAI-2, plasminogen and E-cadherin in A549 cells.** (A) Immunofluorescence images of HAI-2 and E-cadherin in HAI-2-overexpressing A549 cells. Cells were fixed and immunofluorescently stained with anti-HAI-2 mAb (DC16) and anti-E-cadherin pAb (GTX100443, GeneTex). The anti-mouse Alexa Fluor 488 and anti-rabbit Alexa Fluor 568 secondary antibodies were used to recognize their primary antibodies. Nuclei were stained with DAPI. The fluorescent images were captured using a confocal microscope (Leica SP5). (B) Immunofluorescence images of plasminogen and E-cadherin in HAI-2-overexpressing A549 cells. Cells were fixed and stained with anti-plasminogen pAb (GTX102877, GeneTex) and anti-E-cadherin mAb (BD Biosciences). The anti-rabbit Alexa Fluor 488 and anti-mouse Alexa Fluor 568 secondary antibodies were used to recognize their primary antibodies. Nuclei were stained with DAPI. The fluorescent images were captured using a confocal microscope (Leica SP5).

**Supplementary Figure S19**

**
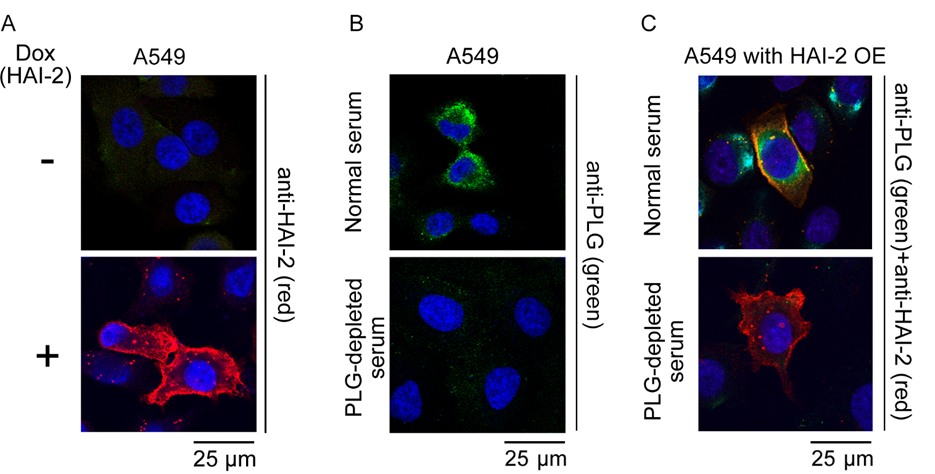
**

**Supplementary Figure S19. Immunofluorescence images of HAI-2 and plasminogen in A549 cells with or without HAI-2 overexpression.** (A) Immunofluorescence images of HAI-2 in A549 cells without/with HAI-2 overexpression after doxycycline induction. Cells were stained with an anti-HAI-2 mAb (DC16). An anti-mouse Alexa Fluor 568 secondary antibody was used to recognize the primary antibody. Nuclei were stained with DAPI. The fluorescent images were captured using a confocal microscope (Leica SP5). (B) Immunofluorescence images of plasminogen in A549 cells. Cells were cultured with normal serum and plasminogen (PLG)-depleted serum. The plasminogen-depleted FBS was prepared and produced after lysine agarose filtration as follows: Ten milliliters of FBS were passed through 1 ml lysine agarose (L5631, Sigma-Aldrich). The filtration was repeated for three times, and the depletion of plasminogen was determined by Western blot. Cells were then incubated with RPMI media supplemented with 10% regular FBS or plasminogen-depleted serum for 24 hours and subjected to fixation. Cells were then stained with anti-plasminogen pAb (GTX102877, GeneTex) and followed by anti-rabbit Alexa Fluor 488 secondary antibody. (C) Immunofluorescence images of plasminogen and HAI-2 in HAI-2-overexpressing A549 cells. A549 cells with HAI-2 overexpression (HAI-2 OE) were cultured with normal serum and plasminogen-depleted serum for 24 hours. After fixation, cells were stained with anti-plasminogen pAb (GTX102877, GeneTex) and anti-HAI-2 mAb (DC16), and followed by anti-rabbit Alexa Fluor 488 and anti-mouse Alexa Fluor 568 secondary antibodies. Nuclei were stained with DAPI. The fluorescent images were captured using a confocal microscope (Leica SP5).

**Supplementary Materials and Methods**

*Cell Culture*- Human lung adenocarcinoma cell lines (PC-9, H1975, CL141, CL97, CL1-0 and CL1-5 cells) were provided by Dr. Pan-Chyr Yang, National Taiwan University Hospital and maintained in RPMI 1640 (Thermo Fisher, MA, USA) supplemented with 10% FBS (Hyclone, GE Life Science, PA, USA) and 1% L-glutamine (Sigma-Aldrich, MO, USA). A549 and H1299 cells (ATCC, VA, USA) were cultured in RPMI 1640 with 10% FBS and 1% L-glutamine. All cells were incubated in a humidified incubator with 5% CO_2_ at 37°C and sub-cultured every 3 days. CL1-5.HAI-2 and control stable pools were generated by transfection of CL1-5 cells with mammalian expression plasmid (pCDNA 3.1 myc/his, Thermo Fisher, MA, USA) and selection of 500 µg/ml G418 (Thermo Fisher, MA, USA) for 2 weeks. Doxycycline-inducible A549 cells for HAI-2 overexpression were established by infection of A549 cells with lentiviral particles carrying tet-aOn gene (tetracycline transactivator, advanced-on; vector: pAS3w.Pbsd, Academia Sinica, Taipei, Taiwan) and HAI-2 cDNA (vector: pAS4w.1.Ppuro, Academia Sinica, Taipei, Taiwan).

*Lentiviral infection for shRNA interference and overexpression-* The lentivirus vector system utilized to deliver the shRNA and gene of interest was purchased from the RNAi Core Facility (Academia Sinica, Taipei, Taiwan) [shHAI-2 (#1 TRC0000073578 and #2 TRC0000073579); shuPA (#1: TRCN0000051088; #2: TRCN0000051092)]. The protocols for viral particle production and infection were provided by the RNAi Core Facility. Briefly, the plasmids for lentivirus packaging and the vectors carrying shRNA/cDNA were co-transfected to HEK293T cells. After 48 hours, the lentiviral particles were produced to the conditioned media and then collected. For infection, 1 ml of virus solution and 2 ml of regular medium were mixed well and added to the cells. Eight micrograms per milliliter (8 µg/ml) of polybrene (Sigma-Aldrich, MO, USA) were supplemented to improve the infection efficiency. Next day the infected cells were refreshed with regular media and cultured for 24 hours. For stable knockdown or gene expression, the cells were selected with 2 µg/ml puromycin (Invitrogen, CA, USA) for at least 2 weeks under regular culture conditions.

*Transwell assay-* For cell invasion assay, 2 µl of Matrigel (Corning, NY, USA) mimicking basement membrane were mixed with 100 ul of autoclaved ddH_2_O and evenly coated on a Boyden chamber (Millipore, CA, USA) by air-dry overnight. The gel was reconstituted with serum-free medium before usage. For cell migration assay, no Matrigel was coated. Equal numbers of cells were seeded into Boyden chambers with 200 µl OPTI-MEM (Thermo Fisher, MA, USA). The chamber was then placed in a well of 24-well dishes, where 700 µl of regular medium was filled to be a chemoattractant in each lower chamber. After incubation for the indicated times, cells were fixed with methanol for 10 min and then stained with 1% crystal violet. The migratory or invasive cells were photographed under a microscope and quantified with ImageJ software 1.50i (NIH, MD, USA).

*Immunohistochemistry (IHC) of human lung cancer tissue arrays-* The protocol of IHC and the tissue array of lung adenocarcinoma were provided by US Biomax Inc (MD, USA). Briefly, the slides of human lung adenocarcinoma tissue microarray (LC641, Biomax) were de-paraffinized by xylene and rehydrated by ethanol. For the frozen slides of xenografted tumor tissues in animals were fixed with cold methanol instead of de-paraffinization. After inactivating peroxidase using 3% H_2_O_2_, the antigen was retrieved by placing the slide in 95°C hot buffer (10 mM sodium citrate, pH 6.0) for 15 min. Tissues were then blocked with 5% goat normal serum, and incubated with primary Ab (DC16: anti-HAI-2 mAb, 1:200, 1% BSA) at 4°C overnight. After three rinses with PBS, secondary Ab conjugated with peroxidase was applied to recognize the primary antibody. After three rinses of PBS, tissues were incubated with DAB (Dako EnVsion, Agilent technologies, CA, USA) to reveal the target proteins according to the vendor’s manual. The Q score of each case in the array was determined by intensity (0-3) multiplying area (1-100) ^2^. The cases with Q score below 150 were defined as low expression, and the others as high expression. The significance of relationship between expression levels and cancer characteristics was determined by chi-square test using Prism software 6 (GraphPad, CA, USA).

*Western blot*- Cells were lyzed with lysis buffer [1% Triton X-100 in PBS (pH 7.0) containing protease inhibitor cocktail (Roche, Switzerland)] and centrifuged at 13,000 r.p.m. at 4°C for 10 min. The supernatants were collected and their protein concentrations were measured by Bradford assay (Bio-Rad, CA, USA). After mixing with 4x SDS sample buffer (8% SDS, 40% glycerol, 0.008% bromophenol blue and 0.25 M Tris pH 6.8, 20% β-mercaptoethanol) and boiling, the samples were subjected to SDS-PAGE and then transferred to PVDF membrane (PerkinElmer, MA, USA). The membranes were blocked with 5% skim milk in TBST buffers and incubated with primary antibodies at 4°C overnight [antibodies: anti-HAI-2 (customized by Kelona, Taipei, Taiwan), anti-c-Myc (Santa Cruz, CA, USA), anti-GST mAb (Yao-Hong Bio, Taipei, Taiwan), anti-plasminogen (GeneTex, CA, USA), anti-urokinase (GeneTex, CA, USA), anti-E-cadherin (BD, NJ, USA), anti-N-cadherin (Merck Millipore, Germany), anti-Vimentin (Proteintech group, IL, USA), anti-Slug (Cell Signaling, MA, USA), anti-pc-MET (Tyr1234/1235) (Cell Signaling, MA, USA), anti-c-MET (Spring Bioscience, CA, USA), anti-phospho-Smad2 (Ser465/467)/Smad3 (Ser423/425) (Cell Signaling, MA, USA), anti-Smad2/3 (Cell Signaling, MA, USA), and anti-β-actin (Sigma-Aldrich, MO)]. M19 and DC16 mAbs were gifts from Dr. Chen-Yong Lin at the Georgetown University, Washington, DC, USA ^1^. After three washes of TBST, the membranes were incubated with secondary antibodies conjugated with HRP (Jackson ImmunoResearch, CA, USA) for 1 hr. Following three washes of TBST, ECL reagent (PerkinElmer, MA, USA) was overlaid on the membrane and the signals were detected by a CCD camera (LAS-4000, Fujifilm, Japan).

*Recombinant protein purification*-GST fusion proteins were produced in BL21 (DE3) *E.coli* using IPTG induction for 4 hrs. After cell lysis, the lysates were collected and the GST fusion proteins are purified by affinity columns (Glutathione Sepharose 4B, GE Life Science, PA, USA). Since GST-KD1 was misfolded in the inclusion body, it was dissolved in 8M urea and refolded by stepwise dialysis before an affinity purification. The recombinant HAI-2 proteins tagged with melittin signal peptide (N terminus) and 6xHis (C terminus) were generated by a baculovirus expression system (Bac-to-Bac system, Thermo Fisher, MA, USA). The titerless amplification of viral particles and production of recombinant proteins were referred to the previous study ^3^. Briefly, High Five cells (Thermo Fisher, MA) were infected with baculoviral particles and cryopreserved (named as baculovirus-infected insect cells, BIIC). Afterwards, the BIICs were thawed and co-cultured with growing insect cells at a ratio of 1:100 for 1-3 days until cell growth stops. The conditioned medium was gathered and fractionated by a cation exchange chromatography (HiTrap SP HP, GE Life Science, PA, USA). The recombinant proteins were then purified form the fractions by using an affinity resin (Talon, GE Life Science, PA, USA).

*GST-pulldown assay and LC/MS/MS*- A549 cells were lyzed with mild lysis buffer [0.5% NP-40, PBS pH 7.2 with protease inhibitor cocktail (Roche, Switzerland)] and collected after centrifugation. For GST pulldown, 10 mg of cell lysates and 10 µg of purified GST-fusion proteins were mixed together and incubated at 4°C overnight. Fifty microliters (50 µl) of pre-washed glutathione beads (GE Life Science, PA, USA) were added into the mixture. The solution was incubated for 30 min. The beads were then collected after centrifugation. After five washes of PBS, the beads were mixed with 50 µl of 1x SDS sample buffer and boiled. The proteins samples were subjected to SDS-PAGE and revealed by SYPRO-Ruby staining (Thermo Fisher, MA, USA) according to the vendor’s manual. For in gel digestion, the gel pieces were washed with 25 mM ammonia bicarbonate and dehydrated by acetonitrile. After rehydration, the reduction and alkylation of the samples were performed by the additions of 10 mM DTT and 55 mM iodoacetamide. The protein samples in gels were then trypsinized [0.1 mg/ml trypsin (Promega, WI, USA)] at 37°C overnight. The digested peptides were extracted by 60% acetonitrile plus 5% folic acid, and subjected to LC/MS/MS analysis for protein identities (Mithra Biotechnology, Inc., Taipei, Taiwan).

*Immunoprecipitation-* Cell lysate was prepared using mild lysis buffer [0.5% NP-40, PBS (pH 7.2) with protease inhibitor cocktail (Roche, Switzerland)]. For immunoprecipitation, 2 mg of cell lysates were incubated with 2 µg of anti-Myc mAbs (Santa Cruz, CA, USA) at 4°C overnight. Ten microliters of protein G-coupled magnetic beads (Protein G Mag Sepharose Xtra, GE Life Science, PA, USA) were added and incubated with the mixture at 4°C for an hour. The beads were collected and washed three times with PBS. The binding proteins were eluted by 30 µl of acid buffer (0.2 M glycine, pH 2.5) and then neutralized by 6 µl of 1 M Tris (pH 8.5).

*Immunofluorescence-* Cells were seeded in 8-well chamber slides (SPL life Science, Korea) and fixed with 4% paraformaldehyde. The samples on the slides were overlaid with blocking buffer (5% normal goat serum, PBS pH 7.2) at R.T. for an hour, and then incubated with primary Abs [1:200 in 1% BSA PBS (pH 7.2)] at 4°C overnight. Following three washes of PBS, the samples were incubated with secondary Abs conjugated with Alexa Fluor [1:500 in 1% BSA PBS (pH 7.2), goat anti-mouse 568 or goat anti-Rabbit 488, Thermo Fisher, MA, USA] at R.T. for an hour. After three washes of PBS, the samples were sealed with Prolong Gold Antifade Reagent (Thermo Fisher, MA, USA). The fluorescent images were captured by a confocal microscope (Leica TCS SP5, Germany). The negative controls for DC16 and anti-plasminogen antibodies were displayed in Supplementary Figure S19.

*Protease activity assay-* For *in vitro* assays, uPA or plasmin proteins were mixed with the indicated concentrations of purified recombinant HAI-2 proteins, and incubated for 30 min at R.T.. Five micro molars (µM) of fluorogenic substrates (Ala-Leu-Lys-AMC for plasmin, Sigma-Aldrich, MO; Gly-Gly-Arg-AMC for uPA, Merck Millipore, Germany) were added into the solution and incubated at 37°C for 30 min. The increased fluorescence (EX:360nm/EM:465nm) generated from the cleavage of substrates was detected by a microplate reader (Paradigm, Beckman Coulter, CA, USA). The approach of cell-surface plasmin activity assay was referred to the previous study ^4^. Briefly, Cells were detached from cell cultures using citric saline (15 mM sodium citrate, 135 mM potassium chloride, pH 7.4). 2x10^4^ cells were suspended in 100 µl PBS (pH7.5) and incubated with 5 µM fluorogenic substrates at 37°C for 30 min. The control experiment was performed in the presence of 20 µg/ml aprotinin (Sigma-Aldrich, MO, USA) to represent background. The fluorescence intensities were measured using a microplate reader (SpectraMax Paradigm, Beckman Coulter, CA, USA), and the actual values of the protease activity were obtained by subtracting aprotinin control.

*Zymography-* The conditioned media of cell cultures were collected and centrifuged to remove cell debris. After mixed with SDS sample buffer [8% SDS, 40% glycerol, 0.008% bromophenol blue and 0.25 M Tris (pH 6.8)], the samples were subjected to SDS-PAGE containing 0.1% gelatin (Sigma-Aldrich, MO, USA). The gel was then washed with 2.5% Triton X-100 (Sigma-Aldrich, MO, USA) and incubated in TNC buffer [50 mM Tris (pH 7.8), 200 mM NaCl, 5 mM CaCl_2_)] at 37°C for 20 hrs. After incubation, the gel was stained with Coomassie Blue (GE Life Science, PA, USA) for 30 min and de-stained with a buffer (40% methanol, 10% acetic acid) for 4 hrs. The images were captured by a CCD camera (LAS-4000, Fujifilm, Japan).

*Lung metastatic assay-* NOD/SCID mice (6 months old) were obtained from the National Laboratory Animal Center (Taipei, Taiwan) breed following the animal use protocol of Academia Sinica IACUC. Cells expressing Luc2 gene were suspended at a density of 1x10^6^ cells per 100 µl OPTI-MEM and intravenously injected into the tail vein of each mouse. To measure the lung cancer metastasis, 200 µl of D-luciferin (15 mg/ml) were intraperitoneally injected to each mouse. The bioluminescence intensity generated from tumor cells was detected at the indicated times by the IVIS imaging system (Xenogen IVIS 50, PerkinElmer, MA, USA). After 10 weeks, the mice were sacrificed and the lungs were cryosectioned for further analysis.

*In situ zymography of plasmin activity-* The *in situ* zymography was performed according to the previous study ^5^. Briefly, the frozen section of a lung tissue was overlaid with 10% polyacrylamide solution [50 mM Tris (pH 7.5), 10% acrylamide/bis-acrylamide (37.5:1), 0.1% ammonia persulfide and 0.04% TEMED] containing 40 µM of fluorogenic substrates (Ala-Leu-Lys-AMC, Sigma-Aldrich, MO, USA) and covered with a coverslip for polymerization at R.T. for 30 mins. The slides were then incubated for the protease action on the substrates at 37°C for an hour. The images were visualized and captured with a fluorescent microscope (Nikon, Japan) using UV excitation (360 nm). The fluorescent intensity was quantified by ImageJ software (NIH, MD, USA).

*Statistics*- The statistical results were calculated from three independent experiments. The significance was determined by one-way ANOVA or Student’s *t*-test using Prism 6 software (GraphPad, CA, USA).

**References:**

1. Benaud C, Dickson RB, Lin CY. Regulation of the activity of matriptase on epithelial cell surfaces by a blood-derived factor. *Eur J Biochem* 2001; **268**(5): 1439-1447.

2. Wu SG, Chang YL, Lin JW, Wu CT, Chen HY, Tsai MF *et al.* Including total EGFR staining in scoring improves EGFR mutations detection by mutation-specific antibodies and EGFR TKIs response prediction. *PLoS One* 2011; **6**(8): e23303; doi 10.1371/journal.pone.0023303.

3. Wasilko DJ, Lee SE, Stutzman-Engwall KJ, Reitz BA, Emmons TL, Mathis KJ *et al.* The titerless infected-cells preservation and scale-up (TIPS) method for large-scale production of NO-sensitive human soluble guanylate cyclase (sGC) from insect cells infected with recombinant baculovirus. *Protein Expr Purif* 2009; **65**(2): 122-132; doi 10.1016/j.pep.2009.01.002.

4. Stillfried GE, Saunders DN, Ranson M. Plasminogen binding and activation at the breast cancer cell surface: the integral role of urokinase activity. *Breast Cancer Res* 2007; **9**(1): R14; doi 10.1186/bcr1647.

5. Nishiuma T, Sisson TH, Subbotina N, Simon RH. Localization of plasminogen activator activity within normal and injured lungs by in situ zymography. *Am J Respir Cell Mol Biol* 2004; **31**(5): 552-558; doi 10.1165/rcmb.2004-0162OC.

**Full Figure Legends**

**Figure 1. Down-regulation of HAI-2 is related to the invasion, survival rate and progression of NSCLC.** Analyses of the cell invasion and the expression of HAI-1 and HAI-2 in different lung cancer cells (A) and in lowly invasive CL1-0 and highly invasive CL1-5 cells (B). Cells were seeded at a density of 5x10^4^ cell per transwell coated with 1 μl Matrigel and incubated for 24 hr. The cells going through the transwell were stained with 1% crystal violet and the numbers of these cells represented the invasive ability (mean ± S.D.; *, *p*<0.05; **, *p*<0.005; ***, *p*<0.001; One-way ANOVA). Cell lysates were subjected to SDS-PAGE and immunoblotting using anti-HAI-1 mAb (M19) and anti-HAI-2 pAb, respectively. β-actin was used as control. (C) Association of survival rate and HAI-2 expression in lung cancer patients. The tissue samples of 255 lung adenocarcinoma patients (SurvExpress, #13 TCGA database) were divided into High risk and Row risk groups according to the HAI-2 expression levels (upper panel). The survival rates of patients in High risk and Low risk groups were analysed and shown in the lower panel. (D) Immunohistochemical images of HAI-2 in lung cancer tissues. The HAI-2 protein levels of lung adenocarcinoma (64 cases, LC641, Biomax) were examined by immunohistochemistry using anti-HAI-2 mAb (DC16) and divided into two groups: high HAI-2 expression (n=28) and low HAI-2 expression (n=36). Two representative images in each group were shown. Scare bar=100 μm. (E) Examination of the associations between HAI-2 protein levels in the IHC images (D) and cancerous characteristics. The significant correlation between HAI-2 expression and cancerous characteristics was statistically determined by Fisher’s Exact test or χ^2^ test.

**Figure 2. Role of HAI-2 in lung cancer cell motility.** (A) Effect of HAI-2 knockdown on A549 lung cell migration and invasion. HAI-2-knockdown (shHAI-2#1 and shHAI-2#2) A549 cells were established using lentiviral infection. The infected cells were selected with 2 μg/ml puromycin for 2 weeks. The HAI-2 protein levels after HAI-2 silencing were analysed by immunoblotting using an anti-HAI-2 pAb. For cell invasion and migration assays, cells were seeded at a density of 5x10^4^ cells per transwell coated with or without 1 μl Matrigel and incubated for 40 and 24 hr, respectively. The cells penetrating through the transwells were stained with crystal violet. The cell numbers were counted and statistically calculated with a ratio to control cells to represent the migrating or invasive abilities (mean ± S.D., n=3, *, *p*<0.05; **, *p*<0.005; ***, *p*<0.001; One-way ANOVA). (B) Effect of HAI-2 silencing on CL1-0 cell migration and invasion. HAI-2-knockdown CL1-0 cells (shHAI-2, clone ID: TRCN0000073578) were set up using lentiviral infection and stable selection using 2 μg/ml puromycin for 2 weeks. shRNAs against luciferase (shLuc) were used as control. Cell lysates were subjected to SDS-PAGE and immunoblot analyses using an anti-HAI-2 pAb. β-actin was used as control. For cell invasion and migration assays, cells were seeded at a density of 5x10^4^ cells per transwell coated with or without 1 μl Matrigel and incubated for 40 and 24 hr, respectively. The cells traveling through the transwells were stained with crystal violet. The cell numbers were counted and statistically calculated with a ratio to control cells. (mean ± S.D., n=3; *, *p*<0.05; Student’s t-test). (C) Effect of HAI-2 overexpression on CL1-5 cell migration and invasion. CL1-5 cells were transfected with pcDNA3.1.HAI-2.myc plasmid or empty vector and the stable pools were selected with 500 μg/ml G418 for 2 weeks. The expression levels of HAI-2 were examined by immunoblotting using anti-HAI-2 pAb and anti-c-Myc mAb (9E10). For cell invasion and migration assays, cells were seeded at a density of 5x10^4^ cells per transwell coated with or without 1 μl Matrigel and incubated for 16 hr. The cells going through the transwells were stained with crystal violet. The cell numbers were counted and statistically calculated with a ratio to control cells (mean ± S.D., n=3; *, *p*<0.05; Student’s t-test). Magnification, x100.

**Figure 3. Identification of HAI-2’s associated proteins in lung cancer cells.** (A) SYPRO Ruby-stained image of HAI-2’s associated proteins in an acrylamide gel after GST-pulldown and SDS-PAGE assays. For GST pulldown assays, 3.2 mg of CL1-5 cell lysates were mixed with 20 μg of GST fusion proteins (GST control, GST-KD1 and GST-KD2). The mixtures were incubated at 4°C overnight. Forty microliters of pre-washed glutathione sepharose beads were added into the mixtures and then incubated at 4°C for 1 hr. The beads were washed with 0.5 ml PBS four times. To elude the associated proteins, the beads were mixed with 100 μl SDS sample buffer with 5% β-mercaptoethanol and then boiled. The eluates were subjected to SDS-PAGE and the gel was stained with 30 ml of SYPRO Ruby protein gel stain overnight at R.T. The signals were captured by a UVP transilluminator. (B) The identity list of GST-pulldown proteins in (A) after LC-MS/MS analysis. (C) Immunoblot analyses of plasminogen after GST-HAI-2’s KD pulldown assays using CL1-5 cell lysate and FBS. One milligram of CL1-5 cell lysates (upper panel) or 100 μl FBS (lower panel) were mixed with 3 μg of GST-fusion proteins (GST control, GST-KD1 and GST-KD2) and then incubated at 4°C for 1 hr. HAI-2’s KD-associated proteins were isolated using 10 μl of pre-washed glutathione sepharose beads and eluted with 30 μl SDS sample buffer. The isolated proteins were analysed by immunoblotting using an anti-PLG (plasminogen) pAb. (D) Co-immunoprecipitation of HAI-2 and plasminogen in HAI-2-overexpressing CL1-5 cells. The lysate of HAI-2-overexpressing CL1-5 cells (1 mg) was mixed with 2 μg anti-c-Myc mAb (9E10) at 4°C for 2 hr. To pull down HAI-2-associated proteins, 10 μl of pre-washed protein G magnetic beads were added into the mixture and incubated at 4°C for 1 hr. After three washes with PBS, the beads were mixed with acid buffer (200 mM Glycine, pH 2.5) and then boiled to elute the captured proteins. The eluted proteins were subjected to SDS-PAGE and immunoblotting using anti-PLG and anti-HAI-2 pAbs. The lysate from control cells was used as control. (E) Interaction of HAI-2 with plasminogen and plasmin using co-immunoprecipitation assays. A549 cells were transduced to carry a HAI-2.tet.aOn gene for HAI-2 overexpression upon doxycycline (Dox) induction. In the presence or absence of 1 μg/ml Dox, cells were incubated with 10 μg/ml of plasminogen (PLG) or plasmin (Plm) for 2 hr in a 37°C CO_2_ incubator. Cell lysates were then collected, mixed with 2 μg anti-c-Myc mAb (9E10) and incubated at 4°C for 2 hr. Ten microliters of pre-washed protein G magnetic beads were added to the mixtures and incubated at 4°C for 1 hr. After three washes with PBS, the beads were mixed with acid buffer (200 mM Glycine, pH 2.5) to elute the products. The eluted samples were subjected to SDS-PAGE and immunoblotting using anti-PLG and anti-HAI-2 pAbs. (F) Subcellular localisation of plasminogen and HAI-2 using confocal microscopy. HAI-2-overexpressing CL1-5 and A549 cells were cultured on cell culture slides for 24 hr. Cells were then fixed with 4% paraformaldehyde, blocked with 5% horse serum and immunocytochemically stained using anti-PLG pAb and anti-HAI-2 mAb (DC16) at 4°C overnight. After three washes with PBS, secondary antibodies (anti-rabbit Ab labelled with Alexa Fluor 488; anti-mouse Ab labelled with Alexa Fluor 568) were applied to samples. After washing, the images were captured by a confocal microscope (TCS SP5, Leical; magnification, x100)

**Figure 4. HAI-2 represses plasmin proteolytic activities and decreases lung cancer cell motility.** (A) rHAI-2 inhibits the proteolytic activity of plasmin rather than uPA. human active uPA (10 nM) or active plasmin (10 nM) was incubated with rHAI-2 (R&D system) at the indicated concentrations for 30 min in 100 μl of PBS (for uPA) or plasmin reaction buffer [50 mM Tris (pH7.4), 100 mM EACA], respectively. Then, 100 μl of artificial substrates (ALK-AMC for plasmin; GGR-AMC for uPA) were added to each solution and incubated at 37°C for 30 min. The fluorescence emitted from the cleaved substrate (EX: 360 nm; EM: 465 nm) was detected by a microplate reader (SpectraMax Paradigm, Molecular Device), statistically calculated and represented as mean ± S.D.. (n=3) (B) Examination of HAI-2 role in uPA-mediated plasminogen activation. Serum-starved A549 cells were treated with or without Doxycycline (1 μg/ml) for HAI-2 overexpression in 6-well dishes. Next day, the medium was refreshed with OPTI-MEM and added with 1 μg/ml human active uPA and 10 μg/ml human plasminogen. After incubation for 6 hr, cell lysates were harvested for SDS-PAGE and immunoblotting using anti-HAI-2, anti-uPA, and anti-PLG Abs. β-Actin was used as control. (C) Inhibitory effect of HAI-2 on the proteolytic activities of uPA-mediated plasminogen activation. Serum-starved A549 cells (1x10^5^ cells) were treated with or without doxycycline (1 μg/ml) for induction of HAI-2 expression. Next day, 100 ng of human active uPA were added into cells and then incubated at R.T. for 30 min. After two washes with PBS, 1 μg of human plasminogen was added to cells and incubated at R.T. for 30 min. The cells were washed twice, mixed with 100 μl of 10 μM artificial substrate (ALK-AMC) and incubated at 37°C for 30 min. The fluorescence emitted from the cleaved substrate (EX: 360nm; EM: 465nm) was detected by a microplate reader (SpectraMax Paradigm, Molecular Device), statistically calculated and represented the plasmin activity. (mean±S.D., n=3, **, *p*<0.005; One-way ANOVA). (D/E) HAI-2 overexpression suppresses plasminogen-induced cell migration (D) and cell invasion (E). After serum starvation and EACA treatment, A549 cells with or without Doxycycline induction (1 μg/ml, overnight) were seeded at a density of 5x10^4^ cells per transwell in the presence and absence of 10 μg/ml plasminogen (PLG). The detailed procedures of cell migration and invasion assays were described in the materials and methods. After 16 hr, the cells traveling through the culture inert under the bottom was stained with crystal violet. The cell numbers were counted, statistically calculated and represented as mean±S.D. (n=3). **p*<0.05; **; *p*<0.005***, *p*<0.001; One-way ANOVA. (F/G) HAI-2 overexpression suppresses plasmin-induced cell migration (F) and invasion (G). After serum-starved and EACA treatment, A549 cells were treated with or without Doxycycline (1 μg/ml) for HAI-2 overexpression. Next day, cells were seeded at a density of 5x10^4^ cells per transwell in the presence/absence of 10 μg/ml plasmin (Plm). The detailed procedures of cell migration and invasion assays were described in the materials and methods. After 16 hr, the cells traveling through the culture inert under the bottom was stained with 1% crystal violet. The cell numbers were counted, statistically calculated and represented as mean±S.D. (n=3). **p*<0.05; **, *p*<0.005; One-way ANOVA). (H) Analysis of cell-surface plasmin activity in HAI-2-overexpressing CL1-5 cells. HAI-2-overexpressing CL1-5 and control cells (Vec) were detached from cell culture dishes with single cell suspension using citric saline. Cells were then suspended in a concentration of 2x10^4^ cells per 100 μl PBS. The equal volume of 10 μM artificial substrate (ALK-AMC) was mixed with 100 μl of cell suspension. The fluorescence (EX: 360 nm; EM: 465 nm) generated during 30 min was recorded by a microplate reader (SpectraMax Paradigm, Molecular Device), statistically calculated and represented as mean±S.D. (n=3). *, *p*<0.05; One-way ANOVA. (I) Effect of HAI-2 knockdown on the cell-surface plasmin activity in A549 cells. HAI-2-silencing A549 and shLuc A549 cells were detached from cell culture dishes using citric saline and suspended in PBS. Cell suspension was prepared with a concentration of 2x10^4^ cells per 100 μl PBS. The equal volume of 10 μM artificial substrate (ALK-AMC) was then mixed with 100 μl of cell suspension in the presence or absence of 50 mM EACA (a plasmin inhibitor). The fluorescence (EX: 360 nm; EM: 465 nm) generated from the plasmin reaction in a 30-minute period was recorded by a microplate reader (SpectraMax Paradigm, Molecular Device), statistically calculated and represented as mean±S.D. (n=3). *, *p*<0.05; **, *p*<0.005; One-way ANOVA. (J/K) Effect of plasmin inhibition on HAI-2 silencing-induced A549 cell migration (J) and cell invasion (K). After serum starvation, cells (HAI-2-knockdown and shLuc A549 cells) were seeded at a density of 5x10^4^ cells per transwell in the presence or absence of 50 mM EACA (a plasmin inhibitor). The detailed procedures of cell migration and invasion were described in the materials and methods. After 24 hr (cell migration) and 40 hr (cell invasion), the cells passing through the transwells were stained with crystal violet. The cell numbers were counted, statistically calculated and represented as mean±S.D. (n=3). *, *p*<0.05; **, *p*<0.005; ***, *p*<0.001; One-way ANOVA. (L/M) Effects of recombinant HAI-2 proteins on the cell-surface plasmin proteolytic activity in CL1-5 (L) and (M) H1299 lung cancer cells. Cells were seeded at a density of 3x10^5^ cells per well in 6-well plates. Next day, the media were refreshed with OPTI-MEM containing the indicated concentrations of rHAI-2 proteins which were purified from a baculovirus expression system. Cell were then detached from the culture dishes using citric saline and suspended in PBS. Then 100 μl PBS containing 2x10^4^ cells were mixed well with 100 μl of 10 μM artificial substrate (ALK-AMC) and incubated at 37°C for 30 min. The fluorescence (EX: 360nm; EM: 465nm) which was generated from plasmin reaction in a 30-minute period was measured using a microplate reader (SpectraMax Paradigm, Molecular Device), statistically calculated and represented as mean±S.D. (n=3). **p*<0.05; **, *p*<0.005; One-way ANOVA). The immunoblot analyses of HAI-2 and uPA after rHAI-2 treatment in CL1-5 and H1299 cells (L/M, lower panel). Cells were seeded at a density of 3x10^5^ cells per well in 6-well dishes in the presence of the indicated concentrations of rHAI-2 proteins and incubated for 2 days. Cells were harvested and lysates were used for immunoblotting using anti-HAI-2 and anti-uPA pAb. β-actin was used as control. (N/O) Effects of rHAI-2 on the migration (N) and invasion (O) of CL1-5 and H1299 cells. Cells were seeded at a density of 2x10^4^ cells per transwell and treated with the indicated concentrations of rHAI-2 proteins. The detailed procedures of cell migration and invasion were described in the materials and methods. The cells passing through transwells were stained with crystal violet, counted, statistically calculated and represented as mean±S.D. (n=3). *, *p*<0.05; **, *p*<0.005; ***, *p*<0.001.

**Figure 5. HAI-2-mediated mesenchymal epithelial transition of NSCLC *via* repressing plasmin activity, HGF/c-Met and TGFβ signalling.** (A) The morphology of CL1-0, CL1-5, and HAI-2-overexpressing CL1-5 cells taken by a microscope. Scale bar= 100 µm. (B/C) Immunoblots of epithelial/mesenchymal cell biomarkers (E-cadherin, N-cadherin, Vimentin and Slug), phospho-c-Met and c-Met in CL1-0, CL1-5, and HAI-2-overexpressing CL1-5 cells. Cell lysates were collected and subjected to SDS-PAGE and immunoblots. β-actin was used as control. (D) The morphology of HAI-2 knockdown (shHAI-2 #1 and #2) and control (shLuc) A549 cells was pictured by a microscope. Scale bar= 100 µm. (E) Immunoblots of HAI-2, uPA, epithelial mesenchymal biomarkers (E-cadherin, N-cadherin, Vimentin and Slug), phospho-c-Met and c-Met in HAI-2 knockdown (shHAI-2 #1 and #2) and control (shLuc) A549 cells. β-actin was used as control. (F) The morphology of A549 cells after the treatment of 10 µg/ml plasminogen or 1 µg/ml doxycycline in a serum-free culture condition for 48 hr. The images were taken by a microscope. Scale bar= 100 µm. (G) Immunoblots of HAI-2, plasmin, epithelial/mesenchymal biomarkers (E-cadherin, N-cadherin, Vimentin and Slug). Cell lysates were subjected to SDS-PAGE and immunoblots. β-actin was used as control. (H) The morphology of HAI-2 or uPA knockdown A549 cells. The images were pictured by a microscope. Scale bar= 100 µm. (I) Immunoblots of HAI-2, uPA, and epithelial/mesenchymal biomarkers (E-cadherin, N-cadherin, Vimentin and Slug) in HAI-2- or uPA-knockdown A549 cells. β-actin was used as control. (J) Effect of plasmin and rHAI-2 on pro-HGF. The role of HAI-2 on plasmin-activated pro-HGF was examined using the following experiment; 100 nM of pro-HGF, plasmin and rHAI-2 proteins were incubated in PBS for 2 hours. Samples were then analysed by immunoblotting using anti-HGF (α-chain specific), anti-plasmin(ogen), and anti-HAI-2 pAbs. (K) The morphology of A549 cells after the treatment of 100 ng/ml pro-HGF or 1 µg/ml doxycycline in a serum-free culture condition for 48 hr (scale bar=100 µm) were pictured by a microscope. Scale bar= 100 µm. (L) Effect of pro-HGF and HAI-2 on the c-Met signalling (phospho-Tyr1234/1235 and total c-Met, treatment for 2 hr) and the biomarkers of epithelial/mesenchymal cells (E-cadherin, N-cadherin, Vimentin and Slug, treatment for 48 hr). Cell lysates were collected and subjected to SDS-PAGE and immunoblots. β-actin was used as control. (M) The morphology of A549 cells after the treatment of 3 µg/ml plasmin, 100 ng/ml pro-TGFβ1 or 1 µg/ml doxycycline in a serum-free culture condition for 48 hr. Before the above treatment, cells were pre-treated with 50 mM EACA for 15 min. The images were pictured by a microscope. Scale bar=100 µm. (N) Immunoblots of HAI-2, plasmin, Smad2/3 signalling [Phospho-Smad2 (Ser465/467)/Smad3 (Ser423/425) and total Smad2/3], and the biomarkers of epithelial/mesenchymal cells (E-cadherin, N-cadherin, Vimentin and Slug) in the presence or absence of pro-TGF, HAI-2 or plasmin in A549 cells. β-actin was used as control. (O) The morphology of HAI-2 knockdown (shHAI-2 #1 and #2) and control (shLuc) A549 cells in the culture media with normal or plasminogen-depleted FBS. Cells were seeded at a density of 1x10^5^ per well in a 6-well dish and cultured in the media with normal or plasminogen (PLG)-depleted FBS for 3 days. Cell morphology and scattering were then captured using a microscope (scale bar=100 nm). (P) Immunoblot analyses of plasminogen, HAI-2, phospho-c-Met, c-Met, E-cadherin, N-cadherin, Vimentin, and Slug in HAI-2 knockdown (shHAI-2 #1 and #2) and control (shLuc) A549 cells in the culture media with normal or plasminogen-depleted FBS. Cells were cultured in the culture media with normal or plasminogen-depleted FBS for 3 days and then the media were refreshed. Three days after the medium refreshment, cell lysates were then harvested for SDS-PAGE and western blot analyses using anti-HAI-2, anti-phospho-c-Met, anti-c-Met, anti-E-cadherin, anti-N-cadherin, anti-Vimentin, and anti-Slug antibodies.

**Figure 6. Down-regulation of HAI-2 increases a lung metastatic ability of A549 cells, the tissue plasmin activity and EMT.** (A) HAI-2-knockdown (shHAI-2) and control (shLuc) A549 cells were intravenously injected into the tail-veins of NOD/SCID mice (1x10^6^ cells per mouse). The metastatic cells in mouse lungs were tracked by bioluminescence and measured by *in vivo* imaging system (Xenogen IVIS 50, Perkin Elmer). The images from three representative mice in each experimental group were shown in week 1, 6 and 9 after tumour injection. (B) The intensities of bioluminescence of metastatic cells were quantified by Living Image Software V4.5.4 and statistically calculated by GraphPad Prism 6 (mean ± SEM., shLuc, n=7; shHAI-2, n=9; *, *p*<0.05; **, *p*<0.005. Student’s t-test). (C) Metastatic lesions in mouse lungs. Mice were scarified in week 10 after tumour injection and the lungs were harvested. The bioluminescence of metastatic cells was visualised by *in vivo* imaging system (Xenogen IVIS 50, Perkin Elmer). (D) Quantification of metastatic lesions in mouse lungs. The bioluminescence of metastatic cells in mouse lungs was quantified by Living Image Software V4.5.4 and statistically calculated by GraphPad Prism 6 (mean ± SEM., shLuc, n=7; shHAI-2, n=9; *, *p*<0.05; **, *p*<0.005. Student’s t-test). (E) *In situ* plasmin proteolytic activity in mouse lungs. The frozen sections of the mouse lung tissues were covered by 10% PAGE containing 40 μM artificial substrates (ALK-AMC) and incubated at 37°C for an hour. The *in situ* plasmin activities were visualised by a fluorescent microscopy (Nikon, Japan) using UV excitation (360 nm). H&E: hematoxylin and eosin stain; Plasmin: *in situ* zymography of plasmin; Phase: phase contrast images. Scare bar= 100 μm. (F) Quantification of *in situ* plasmin proteolytic activity in normal and tumour tissues. The intensities of fluorescence from three different areas in each experiment were taken and statistically quantified by GraphPad Prism 6 as mean ± SD (n=3, **, p<0.01; one-way ANOVA). (G) Immunohistochemical staining of HAI-2, E-cadherin, N-cadherin and Vimentin in the frozen sections of mouse lung tissues. H&E stains were also performed and used as control. Scare bar=100 μm. (H) Quantification of the IHC intensity in four different tumour lesions of each experiment. The intensity of the IHC images was measured and quantified by ImageJ. (mean ± SEM., n=4; *, *p*<0.05; **, *p*<0.005; ***, *p*<0.001, Student’s t-test.) (I) The model of HAI-2-repressing uPAS, EMT, cell invasion and metastasis of lung adenocarcinoma. Plasminogen (PLG) binds to a plasminogen receptor (PLG-R) and uPA binds to uPAR at the plasma membrane of human lung cancer cells. Once PLG and uPA co-localise at lung cancer cell surface, uPA proteolytically activates plasminogen into active plasmin and plasmin feedforward proteolytically activates pro-uPA, which executes a reciprocal activation process to accelerate plasminogen activation for a rapid generation of plasmin at the cell surface. Active plasmin can proteolytically activate pro-HGF, pro-TGF-β1 and MMP-2/9, as well as degrade extracellular matrix (ECM), leading to promoting the EMT, motility and metastasis of human NSCLC. Cell-surface HAI-2 serves as an important suppressor to inhibit membrane-bound plasmin and to block the reciprocal activation of pro-uPA and plasminogen, which result in suppression of the uPA-plasmin system. Through repressing plasmin, HAI-2 reduces the activations of pro-HGF, pro-TGF-β1, MMP-2/9 and ECM degradation, leading to inhibiting the EMT, cell motility and metastasis of human lung cancer cells.
